# A Neutrophil Hijacking Nanoplatform Reprograming NETosis for Targeted Microglia Polarizing Mediated Ischemic Stroke Treatment

**DOI:** 10.1002/advs.202305877

**Published:** 2024-03-05

**Authors:** Na Yin, Wenya Wang, Fei Pei, Yuzhen Zhao, Changhua Liu, Mingming Guo, Kaixiang Zhang, Zhenzhong Zhang, Jinjin Shi, Yun Zhang, Zhi‐Hao Wang, Junjie Liu

**Affiliations:** ^1^ School of Pharmaceutical Sciences Zhengzhou University Zhengzhou 450001 China; ^2^ Henan Key Laboratory of Targeting Therapy and Diagnosis for Critical Diseases Zhengzhou University Zhengzhou 450001 China; ^3^ Collaborative Innovation Center of New Drug Research and Safety Evaluation Zhengzhou University Zhengzhou 450001 China

**Keywords:** drug delivery, ischemic stroke, microglia, neutrophil extracellular traps, neutrophil hitchhiking

## Abstract

Precise and efficient regulation of microglia is vital for ischemic stroke therapy and prognosis. The infiltration of neutrophils into the brain provides opportunities for regulatory drugs across the blood–brain barrier, while hindered by neutrophil extracellular traps (NETs) and targeted delivery of intracerebral drugs to microglia. This study reports an efficient neutrophil hijacking nanoplatform (referred to as APTS) for targeted A151 (a telomerase repeat sequence) delivery to microglia without the generation of NETs. In the middle cerebral artery occlusion (MCAO) mouse model, the delivery efficiency to ischemic stroke tissues increases by fourfold. APTS dramatically reduces the formation of NETs by 2.2‐fold via reprogramming NETosis to apoptosis in neutrophils via a reactive oxygen species scavenging‐mediated citrullinated histone 3 inhibition pathway. Noteworthy, A151 within neutrophils is repackaged into apoptotic bodies following the death pattern reprogramming, which, when engulfed by microglia, polarizes microglia to an anti‐inflammatory M2 phenotype. After four times treatment, the cerebral infarction area in the APTS group decreases by 5.1‐fold. Thus, APTS provides a feasible, efficient, and practical drug delivery approach for reshaping the immune microenvironment and treating brain disorders in the central nervous system.

## Graphical abstract

This study reports an efficient neutrophil hijacking nanoplatform (referred to as APTS) for targeted A151 (a telomerase repeat sequence) delivery to microglia without the generation of neutrophil extracellular traps. APTS provides a feasible, efficient, and practical drug delivery approach for reshaping the immune microenvironment and treating brain disorders in the central nervous system.

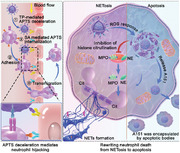

## 1 Introduction

Ischemic stroke, an acute and severe cerebrovascular disease resulting from interrupted cerebral blood flow, is associated with high rates of morbidity, disability, and mortality.^[^ ^1^ ^]^ Currently, the primary approach for ischemic stroke treatment involves thrombolysis or endovascular recanalization therapy to restore blood flow in the affected area.^[^ ^2^ ^]^ However, the pathogenesis of ischemic stroke is greatly influenced by neuroinflammation mediated by activated immune cells following ischemia/reperfusion, which can lead to a persistent neuronal defect or even death.^[^ ^3^ ^]^ Following ischemia/reperfusion, neutrophils accumulate in perivascular spaces and ultimately infiltrate the infarcted brain parenchyma. As the first peripheral immune cells recruited to the ischemic sites, neutrophils play a crucial role in post‐stroke inflammation in the brain.^[^ ^4^ ^]^ The rapid and efficient infiltration of neutrophils into the brain represents both an opportunity and a challenge.

On the one hand, the inflammatory homing properties of neutrophils make them an attractive platform for drug delivery to the brain, such as for ischemic stroke therapy. The blood–brain barrier (BBB), a specialized capillary barrier in the brain, is essential for maintaining homeostasis and protecting the brain from toxic substances circulating in the blood.^[^ ^5^ ^]^ However, the BBB also restricts the delivery of most drugs, to the brain, impeding the treatment multiple of diseases including ischemic stroke.^[^ ^6^ ^]^ Neutrophil‐hitchhiking strategy to overcome BBB has attracted much attention in recent years. For instance, some previous have shown that neutrophils,^[^ ^7^ ^]^ when used as a cellular carrier, can transport puerarin cationic liposomes (L‐Ps) across the BBB, enabling therapeutic molecules to be delivered to the sites of ischemic penumbra.

On the other hand, however, neutrophils can induce strong pro‐inflammatory reactions. For instance, neutrophil infiltration and abnormal activation after ischemic stroke can damage brain tissue and exacerbate inflammation. These adverse effects are often overlooked when developing neutrophils as cellular carriers. Typically, activated neutrophils release nuclear and particulate contents through a unique process called NETosis, forming web‐like DNA structures known as neutrophil extracellular traps (NETs).^[^ ^8^ ^]^ Although NETs, together with fibrin, play a critical role in defending many infectious and non‐infectious pathogens, they also worsen injury by damaging the BBB, promoting the activation of microglia, and increasing inflammation.^[^ ^9^, ^10^ ^]^ DNase I, which cleaves single‐ and double‐stranded DNA, is often used to degrade NETs to suppress immunopathology,^[^ ^11^ ^]^ but it cannot inhibit the NET generation at the source. Moreover, promoting anti‐inflammatory activation of microglia, the brain's innate immune cells,^[^ ^12^, ^13^, ^14^ ^]^ is an effective way to treat ischemic stroke,^[^ ^13^, ^15^ ^]^ however, the feasibility of delivering targeted drugs to microglia in the central nervous system (CNS) remains elusive.

**Figure 1:**
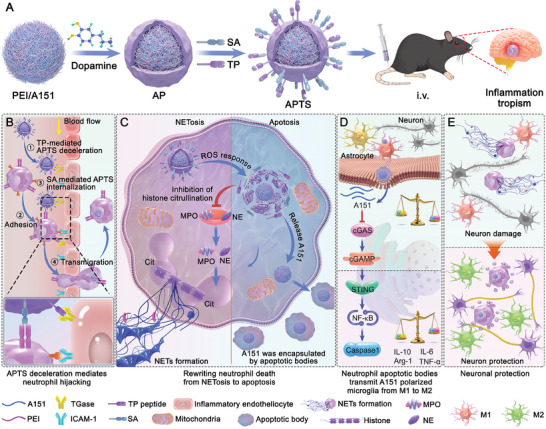
Schematic illustration of a neutrophil hijacking and rewriting nanoplatform for ischemic stroke therapy. A) Preparation process of APTS; B) TP peptide mediated APTS deceleration in inflammatory endothelial cells, triggering the recognition of neutrophils adhered to inflammatory endothelial cells and thereby enhancing neutrophil‐hitchhiking efficiency; C) APTS reprogrammed neutrophil death from NETosis to apoptosis via ROS scavenging‐mediated histone glutamate inhibition pathway, resulting in apoptotic bodies encapsulating A151; D) Apoptotic bodies were efficiently endocytosed by microglia, and the released A151 promoted the transformation of microglia from a proinflammatory M1 phenotype to an anti‐inflammatory M2 phenotype via the cGAS‐STING pathway; E) Neuron protection by rewriting the death pattern of neutrophils and regulating inflammatory microglia.

An ideal treatment strategy for ischemic stroke would involve using neutrophils to both enhance delivery efficiency and reduce neuroinflammation, which, however, remains challenging. In this study, we report a neutrophil hijacking and reprogramming nanoplatform (APTS) for oligodeoxynucleotide (ODN) delivery and neuroinflammation regulation. Specifically, APTS was prepared by modifying polydopamine (PDA)‐coated A151/PEI nanoparticles with targeted peptide (referred to as TP peptide) and sialic acid (SA) (**Figure** **1**A). TGase expressed on the surface of inflammatory endothelial cells facilitated the adhesion of TP peptide as a docking point for APTS, enabling effective recognition and hijacking of neutrophils to the endothelial cells (Figure 1B). APTS‐mediated neutrophil death was reprogrammed from NETosis to apoptosis via reactive oxygen species (ROS) scavenging‐mediated histone glutamate inhibition pathway. Interestingly, the oxidative degradation of PDA by ROS led to the release and integration of A151 (oligonucleotides with telomerase repeat sequences, 5′‐TTAGGGTTAGGTTAGGGTTAGGG‐3′) into apoptotic bodies (ABs), which were subsequently absorbed by microglia (Figure 1C). The absorption of A151 by microglia induced their polarization to an anti‐inflammatory M2 phenotype by inhibiting the cGAS‐STING pathway of microglia (Figure 1D). Reprogramming neutrophils (i.e., NETosis to apoptosis) and reprogramming microglia (i.e., shift to the anti‐inflammatory M2 phenotype) significantly alleviated the overactivated inflammatory environment in the brain by inhibiting the release of NETs and exerted a remarkable therapeutic effect in the middle cerebral artery occlusion (MCAO) model (Figure 1E). Thus, this nanoplatform we developed, which is based on neutrophil hijacking and death pattern reprogramming, offers a new approach to delivering drugs to the brain and managing the overactivated immune microenvironment of brain diseases.

## 2 Results

### 2.1 Preparation and Characterization of APTS

**Figure 2:**
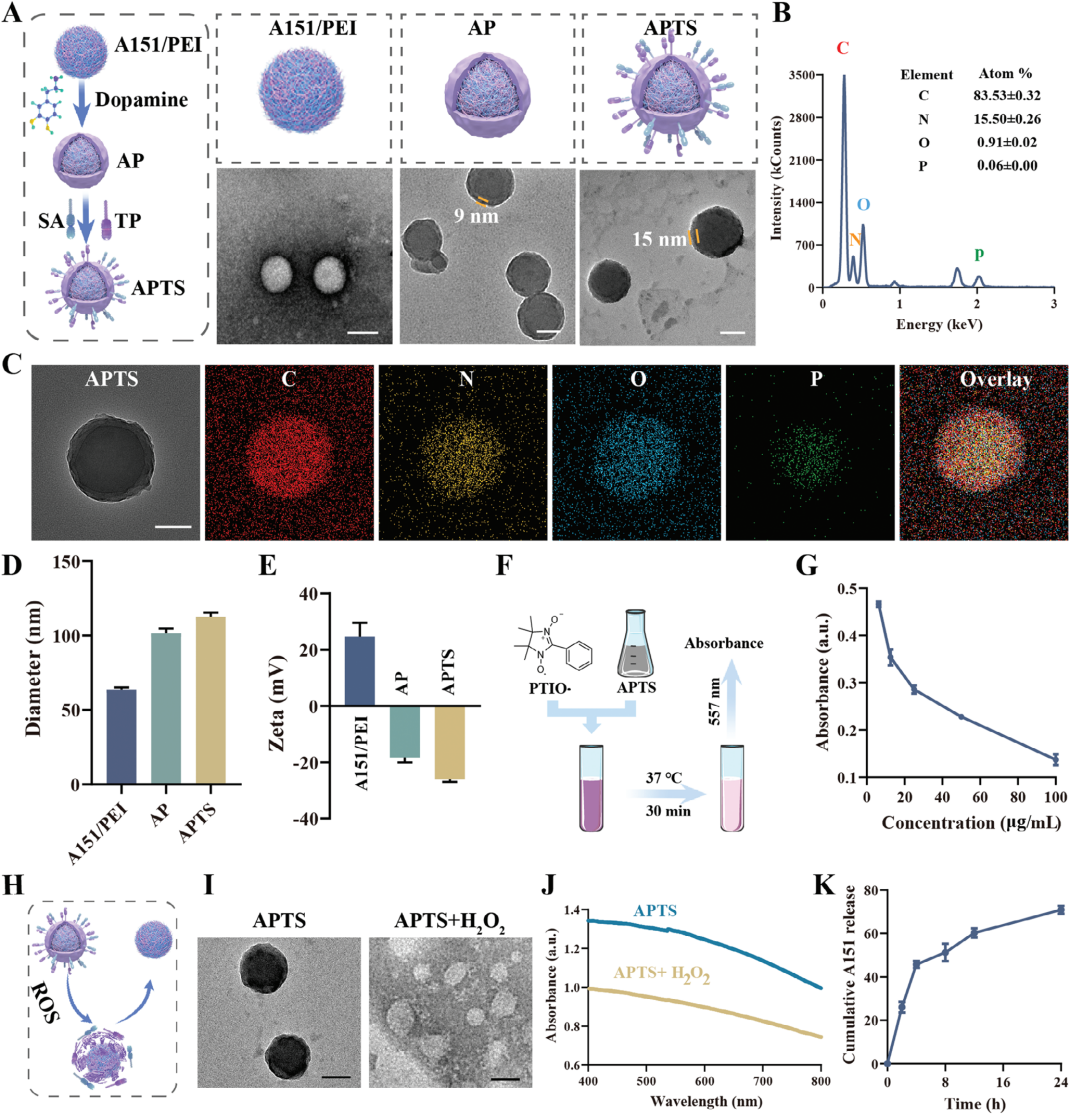
Characterization of APTS NPs. A) Schematic diagrams of different nanoparticles, and negative staining TEM image of A151/PEI, as well as TEM image of AP and APTS. Scale bar: 50 nm. B) Energy‐dispersive X‐ray spectrum of APTS. C) Element mappings of C, N, O, and P elements in APTS. Scale bar: 50 nm. D) Particle size and E) zeta potentials of A151/PEI, AP, and APTS (*n* = 3). F) Schematic diagram of APTS clearing PTIO•. G) Dose‐response curves of APTS in the PTIO•‐ scavenging (*n* = 3). H) Schematic diagram of ROS‐mediated rupture of PDA shell and A151/PEI release. I) TEM images of APTS and negative staining TEM image of APTS treated in H_2_O_2_ solution at 24 h. Scale bar: 50 nm. J) UV–vis absorption spectra and images of APTS treated in H_2_O_2_ solution for 24 h. K) Cumulative release of A151 from APTS at different time points (*n* = 3). Data are presented as mean ± SD.

The APTS was designed with three primary objectives: 1) enhancing neutrophil uptake efficiency and promoting brain enrichment, 2) inhibiting extracellular traps and thus altering the neutrophil death mode, and 3) effectively promoting inflammatory microglia reprogramming for neuronal protection. The synthesis process of APTS is illustrated in **Figure** **2**A. To establish a nanoplatform capable of hijacking and reprogramming neutrophils, the A151/PEI complex was initially integrated into a nano‐complex through the electrostatic binding method previously reported,^[^ ^16^ ^]^ A gel retardation assay was then employed to characterize the A151/PEI complex. With a nitrogen‐to‐phosphate (N/P) ratio of 3 or higher, it was confirmed that A151 could be effectively condensed by PEI at a molecular weight of 10 kDa (Figure S1, Supporting Information). Next, a PDA coating was introduced into the A151/PEI complex, resulting in the formation of A151/PEI@PDA nanoparticles (referred to as AP hereafter). This process was achieved through the oxidative self‐polymerization of dopamine hydrochloride in the tris(hydroxymethyl)aminomethane (tris) buffer (pH 8.5, 10 mm). The successful formation of the PDA layer was indicated by UV–vis absorption at 500–800 nm (Figure S2, Supporting Information). Then, TP‐PEG_5000_‐NH_2_ and SA‐PEG_5000_‐NH_2_ were conjugated to the AP surface (A151/PEI@PDA‐TP/SA, termed the APTS) through Michael addition and/or Schiff base reaction^[^ ^17^ ^]^ (Figure S3, Supporting Information) to promote adhesion to inflammatory endothelial cells and hijack inflammatory neutrophils. Further, the addition of FITC‐labeled SA and Rhodamine‐labeled TP peptide (CGQLKHLEQQEGC) in APTS was analyzed by measuring the fluorescence intensity changes (Figure S4, Supporting Information). The fluorescence intensity before and after modification showed that the modification rate of SA and TP peptide was ≈32.8% and ≈32.3%, respectively.

Transmission electron microscopy (TEM) revealed that all three synthesized nanoparticles, that is, the obtained A151/PEI, AP, and APTS, exhibited a spherical shape and relatively uniform particle size distribution. Compared to A151/PEI, AP displayed a discernible shell–core structure with a PDA shell thickness of ≈9 nm. In comparison, the shell thickness of APTS increased to 15 nm due to the incorporation of dual targeting ligands (Figure 2A). Furthermore, the element analysis and mapping confirmed the elemental compositions of the shell–core structure and the distribution of C, H, O, N, and P (Figure 2B,C). Notably, the P element in A151 was primarily located in the core of APTS, further indicating the successful coating of A151/PEI with PDA. Additionally, as a result of PDA shell coating and modifications with TP peptide and SA, APTS exhibited a significantly increased diameter of 63.8 ± 1.4 nm, compared to 112.5 ± 3 nm for the A151/PEI as measured by dynamic light scattering (DLS), and a decreased zeta potential of −26.0 ± 1 mV, compared to +24.7 ± 5.5 mV for the A151/PEI (Figure 2D,E).

During all stages of ischemic stroke, neutrophils accumulate in the peri‐infarct cortex and generate NETs, exacerbating ischemia/reperfusion injury.^[^ ^18^ ^]^ ROS are essential in the formation of NETs.^[^ ^8^ ^]^ Therefore, the ROS clearance capability of APTS was assessed using 2‐Phenyl‐4,4,5,5‐tetramethylimidazoline‐1‐oxyl 3‐Oxide (PTIO•) radicals (Figure 2F,G), superoxide anions (O_2_^•−^), hydroxyl radicals (•OH), and hydrogen peroxide (H_2_O_2_) (Figures S5,S6, Supporting Information). The results indicate that while being modified, APTS nanoparticles retain a strong antioxidant property.

In our previous study, it was found that PDA could be degraded in the presence of ROS, facilitating efficient cargo release.^[^ ^19^ ^]^ Therefore, the degradation profile of APTS was qualitatively measured using TEM in the presence of H_2_O_2_ (Figure 2H). The representative TEM image in Figure 2I shows morphological changes following APTS degradation, with the PDA shell structure nearly fully degraded at 24 h. In line with this, the UV–vis absorption of APTS at the 400–800 nm range decreased significantly, further confirming the degradation of the PDA shell (Figure 2J); this is consistent with the observed controlled release of A151/PEI (Figure 2K). Moreover, the stability of APTS in both phosphate‐buffered saline (PBS, pH 7.4) and 10% fetal bovine serum (FBS) was examined. The results indicated no significant changes in particle size and zeta potential, demonstrating the high stability of APTS under these conditions (Figure S7A,B, Supporting Information). In addition, the accelerated stability test was carried out at 40 °C and 75% humidity for 4 weeks. The particle size and zeta potential results showed that APTS had excellent stability (Figure S7C, Supporting Information).

### 2.2 APTS Enhances the Efficiency of Neutrophil Hitchhiking

**Figure 3:**
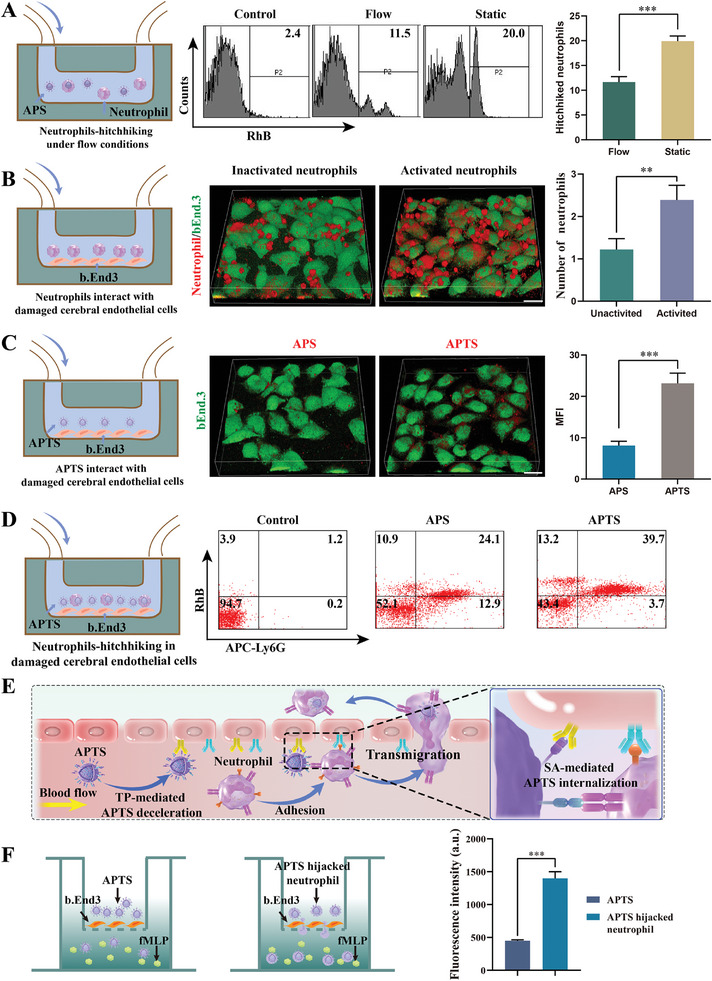
Neutrophils and APTS decelerate in inflammatory endothelial cells to enhance hitchhiking efficiency. A) Schematic diagram of neutrophil‐hitchhiking under flow conditions (left); Flow cytometry was used to evaluate the uptake of APS by neutrophils under flow or static conditions (middle). Proportion of hitchhiked neutrophils (right, *n* = 3). B) Confocal microscopy images showing the adhesion of inactivated and activated neutrophils to endothelial cells. The average number of neutrophils interacted with bEnd.3 cells (*n* = 3). C) Confocal microscopy images showing the adhesion of APS and APTS to bEnd.3 cells. Scale bar: 20 µm. MFI of nanoparticles on bEnd.3 cells (*n* = 3). D) Flow cytometry was used to evaluate neutrophil hitchhiking efficiency in injured endothelial cells. E) APTS deceleration enhanced neutrophil‐hitchhiking schematic diagram. F) Diagram of neutrophils migrating across BBB (left); Quantitative analysis of the in vitro migration (*n* = 3). Data are presented as mean ± SD. Statistical significance was calculated by Student's *t*‐test. **p < 0.01.***p < 0.001.

To assess the specific affinity of APTS for activated neutrophils, *N*‐formyl‐Met‐Leu‐Phe (fMLP) pretreatment was first employed to induce neutrophil activation, which resulted in a significant upregulation of L‐selectin (CD62L) expression on the cell membrane of neutrophils (Figure S8A, Supporting Information). Compared with APTS in inactivated neutrophils, the uptake efficiency of APTS in activated neutrophils increased by 4.6‐fold (Figure S8B, Supporting Information). This observation can be attributed to the specific interaction between SA and the overexpressed CD62L on the membrane of activated neutrophils. These findings further justified the importance of investigating the binding of APS (SA modified A151/PEI@PDA) to activated neutrophils under flow conditions, which can potentially enhance drug delivery. For this, we designed a flow chamber model to evaluate the APS hitchhiking efficiency under flow conditions (**Figure** **3**A). Flow cytometry analysis of circulating neutrophils showed that the uptake efficiency of RhB‐labeled APS by neutrophils under flow conditions was about 1.7 times lower than that of static conditions. This reduction may be due to the decreased recognition efficiency of SA on neutrophils under flow conditions. Therefore, we hypothesize that the complex blood flow environment restricts the hitchhiking efficiency of neutrophils. Also, TP peptide‐modified APTS showed increased hitchhiking efficiency by neutrophils under flow conditions compared with APS (Figure S9, Supporting Information).

Neutrophil‐endothelial interactions play a central role in ischemia/reperfusion injury.^[^ ^20^ ^]^ In our study, the activated and unactivated neutrophils were allowed to circulate in the flow chamber for 1 h. In comparison, activated neutrophils (labeled with red cell tracker) demonstrated substantial aggregation at the site of inflammatory endothelial cells (labeled with green cell tracker) (Figure 3B). The adhesion and deceleration characteristics of neutrophils provided an optimal opportunity for efficient hitchhiking of neutrophils. Inspired by the deceleration effect of neutrophils exerted by inflammatory cerebral vascular endothelial cells, we hypothesized that decelerating nanoparticles in inflammatory cerebrovascular endothelial cells could increase the neutrophil recognition opportunity and enhance the hitchhiking efficiency. TGase, which is upregulated in inflammatory endothelial cells, can catalyze TP peptide adhesion to these cells,^[^ ^21^ ^]^ therefore, TP peptide modification was employed on APS to achieve APTS deceleration. To confirm the upregulation of TGase in inflammatory endothelial cells, mouse brain endothelial cells (bEnd.3) were first stimulated with anti‐tumor necrosis factor‐α (TNF‐α) to mimic inflammation. Enzyme‐linked immunosorbent assay showed that the expression of inflammatory factors interleukin‐6 (IL‐6) and monocyte chemoattractant protein‐1 (MCP‐1) increased by ≈1.6 and 1.4 folds, respectively (Figure S10A, Supporting Information), indicating successful induction of inflammatory endothelial cells. As expected, TGase expression was also significantly upregulated in these cells (Figure S10B,C, Supporting Information). Moreover, compared to APS, modification with TP peptide enhanced the adhesion of APTS to bEnd.3 cells (Figure 3C). Flow cytometry analyses revealed that the neutrophil hitchhiking efficiency of APTS was improved by 1.7‐fold compared to APS (Figure 3D), suggesting that the deceleration of APTS and neutrophils at the site of inflammatory endothelial cells boosted the hitchhiking neutrophils efficiency (Figure 3E). Moreover, neutrophil hitchhiking of APTS had no significant impact on neutrophil activity (Figure S11, Supporting Information).

Transwell migration analysis showed that APTS‐loaded neutrophils displayed fMLP‐activated chemotaxis comparable to that of blank neutrophils (Figure S12A,B, Supporting Information). More importantly, RhB‐labeled APTS remained distributed in migrated neutrophils, which was critical for the efficient delivery of APTS to the ischemic area (Figure S12C, Supporting Information). Quantitative analysis demonstrated that the fluorescence signals of APTS‐hijacked neutrophils in the basal chamber were 3.1‐fold greater than those of the APTS group (Figure 3F). Taken together, these results suggest that neutrophils could still detect inflammatory signals and migrate to inflammatory sites after being loaded with APTS. Fluorescence resonance energy transfer (FRET) is a powerful technique for measuring molecular distance at the nanoscale.^[^ ^22^ ^]^ When the sequence of A151 remained intact, non‐radioactive energy transfer and fluorescence quenching of FAM fluorophore were observed. Enhanced fluorescence intensity indicated that DNaseI could catalyze A151 cleavage (Figure S13A,B, Supporting Information). In neutrophils, compared to the DNaseI + A151 group, the APTS group showed almost no green fluorescence signal, indicating that A151 was relatively stable within cells (Figure S13C, Supporting Information).

### 2.3 APTS Transforms NETosis to Apoptosis in Neutrophils

**Figure 4:**
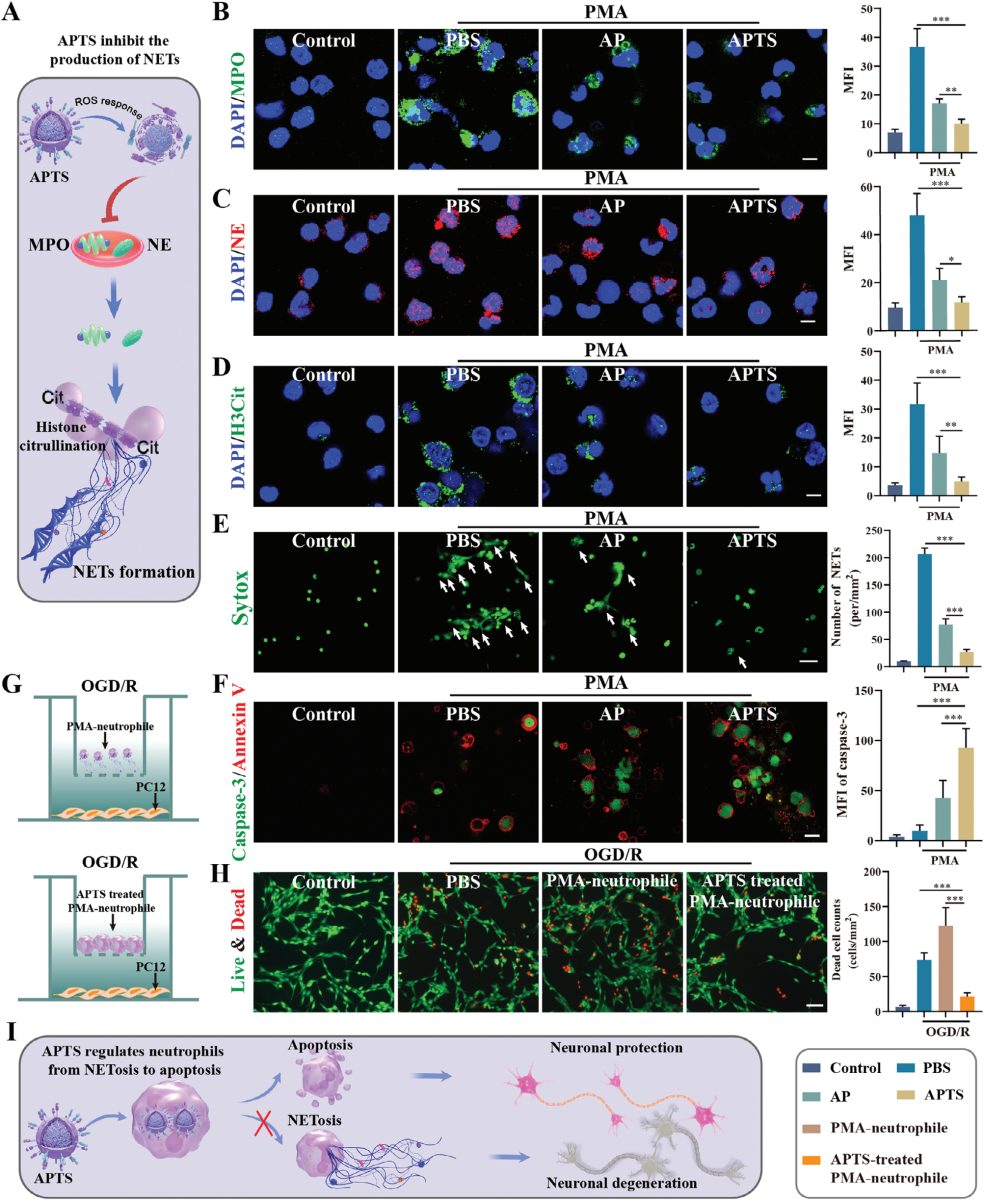
APTS regulates neutrophils from NETosis to apoptosis. A) Schematic diagram of APTS inhibiting the production of NETs. B) Representative images of MPO (green) in neutrophils. Nuclei were visualized with DAPI (blue). Neutrophils were induced by 100 nm phorbol 12‐myristate 13‐acetate (PMA) for 4 h and then treated with PBS, AP, and APTS. Scale bar: 5 µm. Quantitative analysis relative fluorescence intensity of MPO (*n* = 6). C) Representative images of NE (red) in neutrophils. Nuclei were visualized with DAPI (blue). Neutrophils were induced by 100 nm PMA for 4 h and then treated with PBS, AP, and APTS. Scale bar: 5 µm. Quantitative analysis relative fluorescence intensity of NE (*n* = 6). D) Representative images of H3Cit (green) in neutrophils. Nuclei were visualized with DAPI (blue). Neutrophils were induced by 100 nm PMA for 4 h and then treated with PBS, AP, and APTS. Scale bar: 5 µm. Quantitative analysis relative fluorescence intensity of H3Cit (*n* = 6). E) Representative confocal laser scanning microscopy (CLSM) image of neutrophil extracellular DNA (Sytox, green). Arrows indicate extracellular DNA fibers. Scale bar: 25 µm; The number of neutrophil NETs with different treatments (*n* = 6). F) The apoptosis images of neutrophils cells detected by annexin V‐mCherry/caspase‐3 double‐label technique. Scale bar: 10 µm. Quantitative analysis relative fluorescence intensity of caspase‐3 (*n* = 6). G) Diagram of co‐culture of neutrophils and PC12 in transwell dishes. PMA‐neutrophils were pretreated with or without APTS and then co‐cultured with PC12 cells. H) Live & Dead staining for PC12 cells. Scale bar: 200 µm; Quantification of dead cell numbers in different treatments (*n* = 6). I) Schematic diagram of the regulation of neutrophil death pattern and neuron protection. Data are presented as mean ± SD. Statistical significance was calculated by one‐way ANOVA. *p < 0.05, **p < 0.01, ***p < 0.001.

Following an ischemic stroke, a robust inflammatory response occurs, leading to the rapid recruitment of neutrophils.^[^ ^23^ ^]^ These neutrophils release NETs, which contain a large number of pro‐inflammatory factors and exacerbate nerve damage.^[^ ^9^ ^]^ Intracellular ROS is known to prompt the release of granular proteins, like myeloperoxidase (MPO) and neutrophil elastase (NE), as well as histone H3 citcitylation (H3Cit), all of which are essential for NETs formation (**Figure** **4**A).^[^ ^24^ ^]^ Therefore, we assessed APTS's ability to scavenge ROS. The results suggested that APTS could effectively clear intracellular ROS, •OH, O_2_^•−^, and H_2_O_2_ (Figure S14, Supporting Information). Furthermore, we observed a reduction in MPO, NE, and H3Cit (Figure 4B–D). H3Cit can weaken the electrostatic interaction between histone and DNA, leading to chromatin decondensation, which is often a marker of NET formation. We used the fluorescent dye Sytox to validate the diminished production of NETs by observing the formation of extracellular DNA fibers. In line with our hypothesis, APTS treatment led to a 7.7‐fold decrease in extracellular DNA fiber production (Figure 4E). Interestingly, we found that following inhibition of NET formation in neutrophils by APTS, phosphatidylserine (PS) (Annexin V, a PS binding protein)^[^ ^25^ ^]^ was exposed to the cell surface (Figure 4F). In healthy cells, PS is predominantly confined to the inner plasma membrane leaflet. During apoptosis, externalized PS is commonly regarded as a phagocytosis signal of dying cells.^[^ ^26^ ^]^

To further confirm the conversion from NETosis to apoptosis in neutrophils, we used Annexin V and caspase‐3 to double‐label of neutrophils. In comparison to the PBS group, the APTS group exhibited a 9.5‐fold increase in the caspase‐3 activity (Figure 4F). We then evaluated the impact of NETs on neuronal viability using the oxygen‐glucose deprivation/reoxygenation (OGD/R) model (Figure 4G). Live/dead staining results demonstrated that neutrophil NET formation (PMA‐neutrophile) significantly aggravated neuronal damage, whereas APTS‐treated PMA‐neutrophile promoted neuronal survival (Figure 4H). The dead neurocyte of the APTS group had a 5.8‐fold decrease compared with the PMA‐neutrophile group. Thus, APTS can inhibit NET formation and mitigate the detrimental effects of NETs on ischemic neurons. The overall process of APTS modulating neutrophils from NETosis to apoptosis and protecting neurons is summarized in Figure 4I.

### 2.4 Apoptotic Body‐Mediated Delivery of Gene‐Drugs Modulates Microglial Inflammation

**Figure 5:**
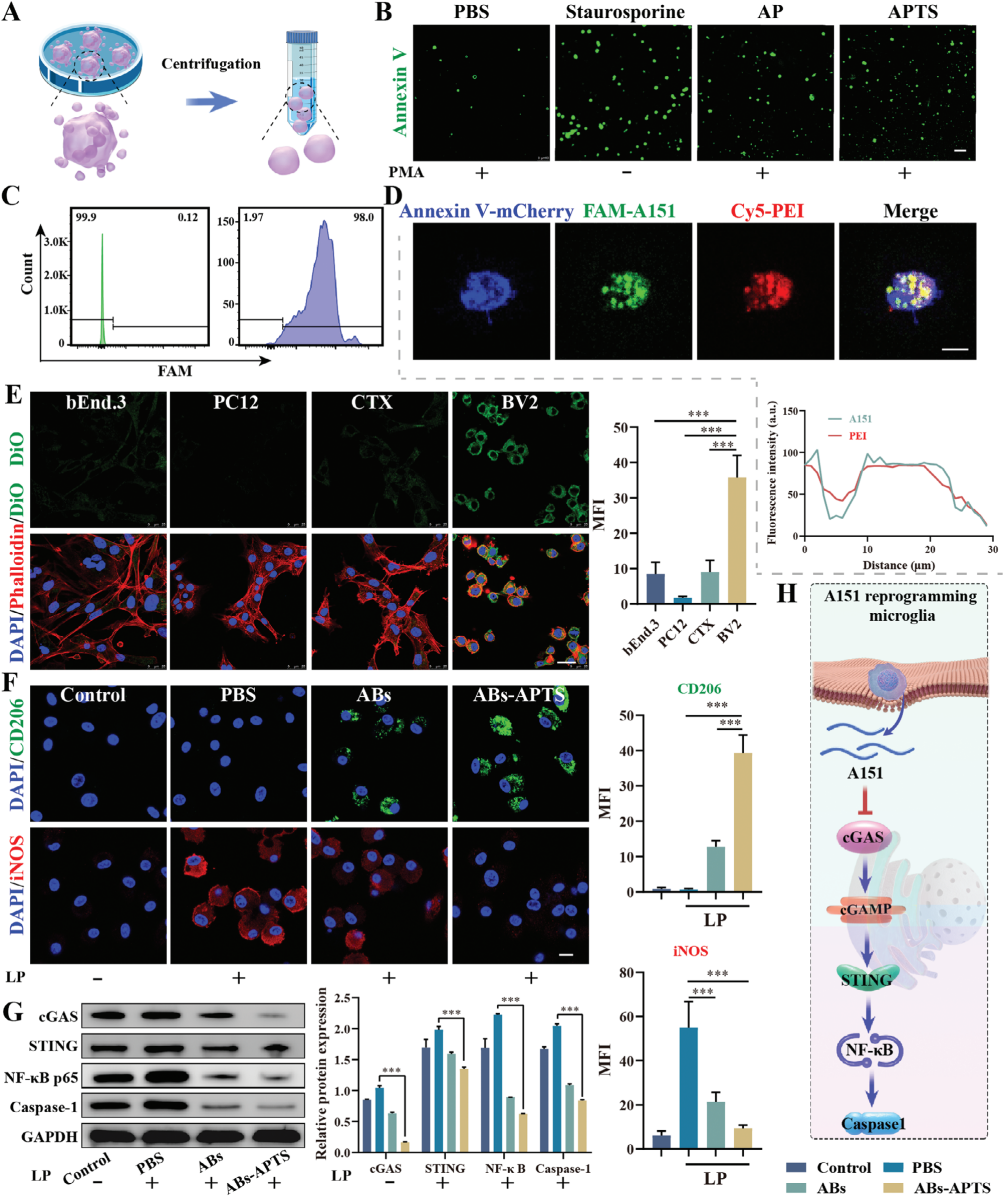
Apoptotic body delivery ODN regulates microglial inflammation. A) Schematic diagram of ABs extraction. B) Annexin V‐FITC of ABs. Scale bar: 20 µm. C) Flow cytometry analysis of A151 in ABs. D) Evaluation of A151 morphology in ABs by CLSM. Scale bar: 5 µm. Colocalization analysis of PEI and A151. E) The uptake of DiO‐labelled ABs in bEnd.3, PC12, CTX and BV2 cells. Scale bar: 25 µm; Quantitative analysis of ABs uptake in bEnd.3, PC12, CTX and BV2 group (*n* = 6). F) Representative fluorescence images of the microglia phenotypes and the quantitative analysis of the CD206/iNOS (*n* = 6). Scale bar, 10 µm. G) Western blot analysis and quantification of cGAS, STING, NF‐κB p65, and Caspase‐1 of BV2 cells after different treatments (*n* = 3). H) Schematic representation of A151 inhibiting the cGAS‐STING pathway. Data are presented as mean ± SD. Statistical significance was calculated by one‐way ANOVA. ***p < 0.001.

APTS within neutrophils can be encapsulated by apoptotic bodies. Immunofluorescence microscopy revealed that APTS suppressed the formation of NETs, and almost no nanoparticles were leaking from the cells. In contrast, liposome nanoparticles lacking NETs‐scavenging capacity were released into the extracellular space along with NETs (Figure S15, Supporting Information). Following neutrophil apoptosis, ABs were isolated using differential centrifugation (**Figure** **5**A). To confirm the successful extraction of ABs, Annexin V was detected by immunofluorescence staining, while cleaved caspase‐3 was detected by western blot (Figure 5B and Figure S16A, Supporting Information). The small size (1–5 µm) of ABs made them easily distinguishable from cell bodies, and a high proportion of ABs production, ≈67.4%, was observed in apoptotic neutrophils by flow cytometry (Figure S16B, Supporting Information). To track the distribution of A151 in ABs, A151 was labeled with FAM. The successful encapsulation of A151 in ABs was verified by flow cytometry (Figure 5C). Similarly, the distribution of A151 and PEI in ABs was evaluated using FAM labeled A151 and Cy5 labeled PEI. A high degree of co‐localization between the A151 and PEI signals was observed, indicating stable A151/PEI encapsulation during AB production (Figure 5D). Subsequently, the uptake of ABs by various cells cell types, including mouse cerebral vascular endothelial cells (bEnd.3), neuron‐like PC12 cells, astrocytes cells (CTX), and mouse microglia cells (BV2), was evaluated. The results showed that AB uptake in BV2 cells was significantly higher than that in other cell types (Figure 5E).

To investigate the potential impact of A151‐loaded ABs on the polarization of microglia in vitro, BV2 were treated with LPS and poly (dA:dT) (referred to as LP), a synthetic dsDNA analog to induce inflammation, followed by the addition of PBS, ABs, and APTS (ABs obtained after treatment of neutrophils with APTS) to the culture system, using unstimulated BV2 as a control. CLSM results demonstrated that LPS induced the M1 phenotype (iNOS^+^) in BV2 cells, which was significantly reduced following APTS treatment. Concurrently, CD206 (M2 marker) expression in the APTS group significantly increased (Figure 5F). Moreover, various pro/anti‐inflammatory cytokine (e.g., IL‐6, TNF‐α/IL‐10, and Arg‐1) levels were observed to decrease or increase at 48 h after APTS treatment (Figure S17, Supporting Information). Additionally, we found that LP‐treated BV2 cells (BV2‐M1) exacerbated neuronal damage under OGD/R conditions. Interestingly, BV2 cells post ABs‐APTS reprogramming showed a significant increase in neuron numbers (Figure S18, Supporting Information). To further investigate the mechanism by which APTS regulates microglia phenotypic changes, western blot experiments were used to assess the expression of proteins in related pathways. APTS down‐regulated cGAS, STING, NF‐κB p65, and caspase‐1, compared to control groups (Figure 5G). Collectively, these findings suggest that APTS can inhibit cGAS‐STING signaling and prevent NF‐κB activation, ultimately attenuating caspase 1‐induced pyroptosis, thereby reducing the transcription of pro‐inflammatory cytokines and chemokines (Figure 5H).

### 2.5 Distribution of APTS in Ischemic Sites

**Figure 6:**
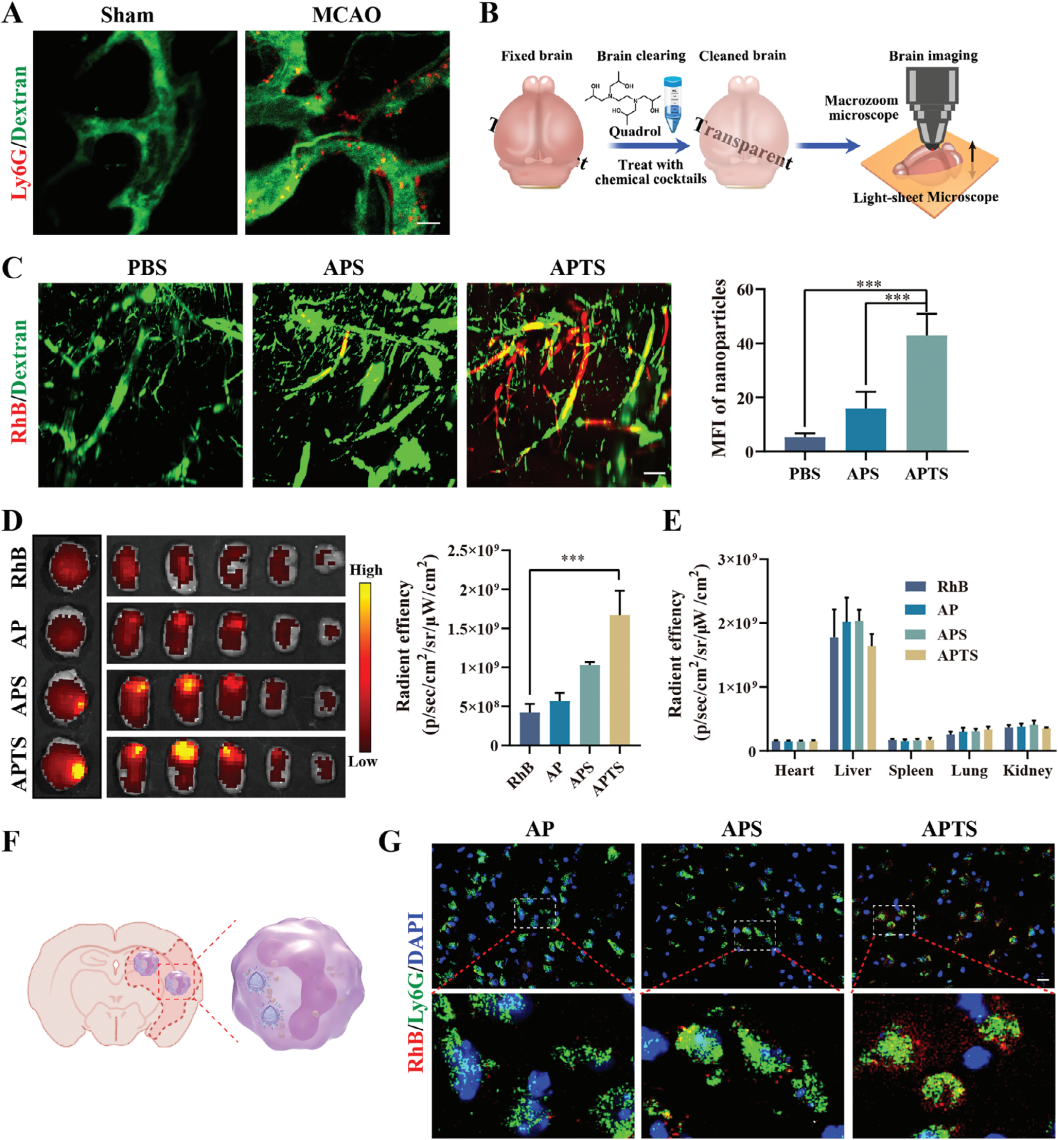
In vivo distribution of nanoparticles. A) Representative in‐vivo multiphoton microscopy images of neutrophils (red) and brain vessels (green) of mice at 3 days. Green: FITC dextran‐labeled blood vessels; Red: Neutrophils were labeled by PE‐conjugated monoclonal Ly6G antibody. Scale bar: 50 µm. B) Schematic diagram of nanoparticle adhesion imaging at the vascular site. C) Representative light‐sheet microscope images of the distribution of nanoparticles in the vascular endothelium and corresponding quantitative fluorescence analysis (*n* = 6). Green: FITC dextran‐labeled blood vessels; Red: RhB‐labeled APS or APTS. Scale bar: 200 µm. D) Representative ex vivo brain fluorescence images and corresponding quantitative fluorescence analysis of the brain and E) major organs dissected from MCAO mice at 12 h after intravenous injection (i.v.) with AP, APS, or APTS (*n* = 3). F) Schematic diagram of the distribution of nanoparticles in brain neutrophils. G) Distribution of AP, APS, or APTS in the ipsilateral brain 24 h after injection (blue: DAPI; green: neutrophils; red: nanoparticles). Data are presented as mean ± SD. Statistical significance was calculated by one‐way ANOVA. ***p < 0.001.

To validate the distribution of APTS in vivo, a mouse brain ischemia/reperfusion injury model was established using the MCAO method. Laser speckle contrast imaging confirmed successful cerebral ischemia and reperfusion (Figure S19, Supporting Information). Using an anti‐MPO antibody, we detected increased infiltration of neutrophils following ischemic stroke (Figure S20, Supporting Information). The adhesion and extravasation of neutrophils on blood vessels in the brains of ischemic mice were further observed using in vivo multiphoton microscopy (**Figure** **6**A). Specifically, to assess APTS adhesion to vascular endothelial cells in vivo, mice were injected with RhB‐labeled nanoparticles (APS or APTS). The brain tissue was then collected and processed transparently (Figure 6B). The results showed that modification of TP peptide resulted in APTS adhering to endothelial cells (Figure 6C), and the distribution of APTS at the vascular site over time increased within 40 min (Figure S21, Supporting Information). The distribution of RhB‐labeled AP, APS, and APTS was also monitored based on the fluorophores (Figure 6D,E and Figure S22, Supporting Information). The ex vivo brain fluorescence signal in the APTS group was four times that of the PBS group. We then observed enhanced APTS within neutrophils (Figure 6F,G), suggesting that nanoparticle‐carrying neutrophils facilitated BBB penetration. Moreover, various doses of APTS (5, 10, and 15 mg kg^−1^) were administered to healthy mice for in vivo toxicity evaluation. After 14 days, the mice were sacrificed to obtain the heart, liver, spleen, lung, kidney, and brain tissues for hematoxylin and eosin (H&E) staining, and blood was collected for a routine blood test. H&E staining (Figure S23, Supporting Information) and blood routine parameters (Figure S24, Supporting Information) revealed no abnormalities in groups with different APTS doses when compared to the control group, indicating that APTS had no obvious pathological toxicity in vivo.

### 2.6 Therapeutic Effect of APTS In Vivo

**Figure 7:**
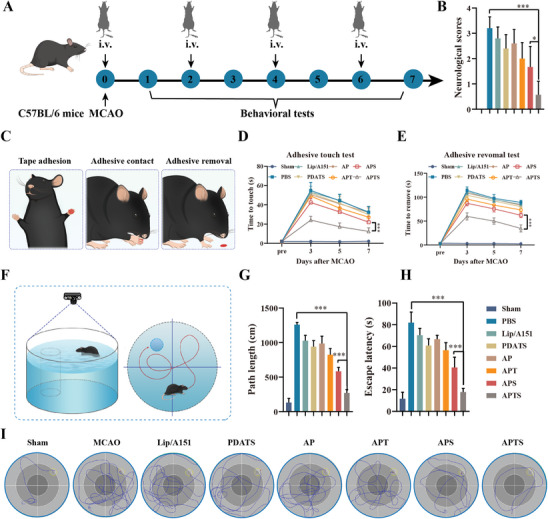
In vivo neurological functional recovery after stroke by APTS treatment. A) Schematic diagram of the treatment protocol in mice (*n* = 8). B) Neurological scores. A higher score indicates a more severe injury. C) Schematic illustration of the adhesive test. D) Time to touch and E) removal of the tape of different treatment groups. Two‐way ANOVA was used to calculate *p*‐values. F) Schematic illustration of Morris water maze test. G) Path length and H) escape latency of mice after different treatments. I) Representative swimming track trails in Morris Water Maze. Values represent mean ± SD. **p < 0.01. ***p < 0.001.

Behavioral tests serve as a measure of neurological function. To evaluate the neurological functions of MCAO mice following APTS treatment, neurological deficit scores, adhesive‐removal tests, and Morris water maze experiments were conducted (**Figure** **7**A). APTS treatment significantly improved neural function scores in mice compared with those treated with PBS or APS (Figure 7B). On the 7th day post‐stroke, the time taken to contact and remove tape in the adhesion test for stroke mice treated with APTS was significantly shorter than for those treated with PBS or APS (Figure 7C–E). To further corroborate the therapeutic effect of APTS, the spatial cognitive function of mice treated with APTS was assessed using the Morris water maze. After treatment, the mice were subjected to the Morris water maze to record their swimming trajectories. Compared to the PBS group, the path length of APTS‐treated mice decreased markedly. Similar findings were also observed in the escape latency test, indicating that the learning and memory functions of mice were improved after APTS treatment (Figure 7F–I).

**Figure 8:**
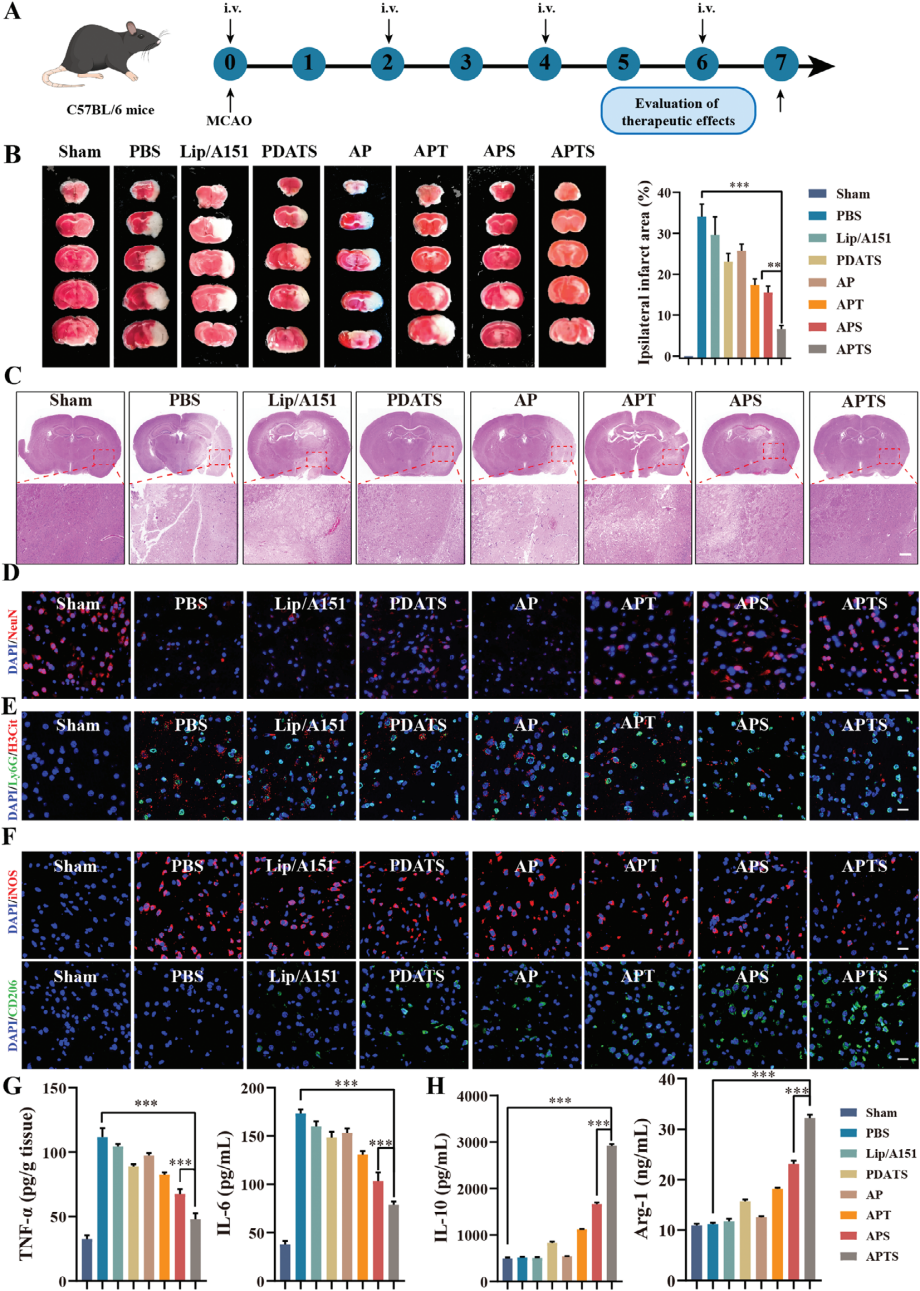
Therapeutic efficacy of APTS for ischemic stroke. A) Treatment regime of mice (*n* = 8). B) Representative TTC staining images of brain slices treated with different formulations. The brain was dissected into 2 mm slices and stained with 2% TTC for 20 min at 37 °C. Quantification of the infarct area (data presented as the percentage of white infarct area, *n* = 3). C) Representative H&E staining images of the pathological examination of MCAO mice cerebral sections. D) Representative images of NeuN staining (red, representing neurons). DNA was stained with DAPI (blue). Scale bar: 20 µm. E) Representative images of Ly6G (green, representing neutrophils) and H3Cit (red, representing NETs) staining. DNA was stained with DAPI (blue). Scale bar: 20 µm. F) Representative images of iNOS (M1 microglia marker) and CD206 (M2 microglia marker) (red: iNOS; green: CD206; blue: DAPI; scale bar: 20 µm) to determine microglia phenotype treated with different formulations. G,H) Expression of TNF‐α, IL‐6, IL‐10, and Arg‐1 in the ischemic brain after different treatments (*n* = 3). Data are presented as mean ± SD. Statistical significance was calculated by one‐way ANOVA. **p < 0.01. ***p < 0.001.

Following the treatments, the mice were sacrificed to evaluate the therapeutic effect (**Figure** **8**A). The protective effect of APTS was evaluated by staining the infarct area with triphenyl tetrazolium chloride (TTC) (Figure 8B). The white area represents the infarct region. TTC staining showed that the PBS group had the largest infarct area while APTS treated group had the smallest infarct area. While the cerebral infarction area in the APTS group decreased by 5.1‐fold compared with the PBS group. H&E staining also demonstrated a prominent brain protective effect of APTS compared to other groups (Figure 8C). To assess BBB permeability, Evans Blue (EB) was administered intravenously (Figure S25, Supporting Information). Compared to other groups, APTS significantly alleviated EB extravasation exhibiting the strongest post‐stroke BBB repair effect. In line with this, staining of NeuN, a neuron‐specific nuclear protein marker, indicated staining results showed that APTS significantly protected neurons from apoptosis compared to other groups (Figure 8D and Figure S26A, Supporting Information). Furthermore, APTS was found to significantly reduce neutrophil NETs by decreasing H3Cit levels in these neutrophils (Figure 8E and Figure S26B, Supporting Information). In addition, A151 further alleviated inflammation by shifting microglia from a pro‐inflammatory to an anti‐inflammatory phenotype, with a significant increase in the fluorescence signal of the anti‐inflammatory marker CD206 and a significant decrease in that of the pro‐inflammatory marker iNOS (Figure 8F and Figure S26C, Supporting Information). The mitigated inflammation was also confirmed by decreased expression of the pro‐inflammatory cytokines IL‐6 and TNF‐α, and increased expression of the anti‐inflammatory cytokines Arg‐1 and IL‐10 (Figure 8G,H). Moreover, histological examination of the major organs revealed no discernible organ damage 7 days after APTS treatment, indicating no potential in vivo toxicity caused by APTS (Figure S27, Supporting Information).

## 3 Discussion

We developed an efficient nano‐immunosuppressive platform for hitchhiking neutrophils in vivo by harnessing the physiological characteristics of native neutrophils to enhance neuroinflammatory regulation potential. Unlike traditional nanoparticles that hitchhike neutrophils in flowing blood circulation, APTS can be anchored to injured vascular endothelial cells via TGase, thus improving neutrophil hitchhike efficiency. The simultaneous affinity adhesion of neutrophils and nanoparticles to injured blood vessels significantly increased the probability of nanoparticles being recognized and engulfed by neutrophils. This enhanced hitchhiking efficiency due to deceleration provides a novel strategy for targeted drug delivery in vivo, which has never been reported previously.

In this study, APTS uptake by neutrophils facilitated neutrophil reprogramming from NETosis to apoptosis through the ROS scavenging mediated citrulline histone inhibition pathway. A151 was encapsulated by apoptotic bodies and specifically engulfed by microglia. The exposure of phosphatidylserine on the surface of apoptotic bodies played an important role in microglial cell recognition and phagocytosis. For instance, the uptake of apoptotic bodies by BV2 cells was 4.2, 20, and 4 times that of bEnd.3, PC12, and CTX cells, respectively. Enhanced ODN delivery safely and effectively regulated microglia via the cGAS‐STING pathway. For the first time, we utilized the natural transport properties of neutrophils while overcoming their adverse effects on the brain, providing a powerful tool for targeted microglial delivery in a variety of brain diseases.

Therefore, a neutrophil hijacking nanoplatform reprograming NETosis was developed for ODN delivery and neuroinflammation regulation, which can be used to treat ischemic stroke effectively. APTS can not only mediate the enhancement of neutrophil hitchhiking efficiency through effective deceleration of inflammatory vascular endothelial cell sites but also inhibit NET production, reprogram neutrophils for apoptosis, and prevent NET‐mediated neuronal damage. Remarkably, apoptotic bodies produced by apoptotic neutrophils could effectively encapsulate A151 and enable accurate and effective delivery of drugs to microglia, enhancing the capacity of APTS in regulating inflammation. In the MCAO mouse model, the nanoplatform can effectively reduce neuroinflammation and infarct volume. Thus, this work revealed a novel drug delivery approach and provided a powerful tool for treating inflammatory diseases in the CNS.

## 4 Experimental Section

### Study Design

The objective of this study was to rationally develop an immunosuppressive nanoplatform (APTS) with high‐performance hitchhiker neutrophils promoting neuroinflammatory regulation and to verify the therapeutic effect of this strategy in MCAO mice. First, APTS was prepared and characterized their morphology, ROS scavenging capacity, and oxidative degradation characteristics. Second, the efficiency of APTS in hijacking neutrophils in the flow chamber model, the mechanism of inhibiting NET production, the ability of microglial reprogramming, and neuronal protection were investigated. Then, distribution experiments in vivo were performed to detect the ability of the nanoplatform to hijack neutrophils across the BBB to achieve brain enrichment. Last, the MCAO model was used to analyze the inflammatory regulation and neuroprotective effect of APTS.

### Preparation of AP

10 OD A151 was dissolved in ultrapure water, mixed with polyethyleneimine (PEI) at a nitrogen‐to‐phosphate (N/P) ratio of 3, incubated for 30 min at 25 °C to form A151/PEI complex, and centrifuged at 30 kD at 12 000 rpm for 30 min. Then, A151/PEI complex was added to 1 mL Tris‐HCl (pH 8.5, 10 mm) buffer of dopamine hydrochloride (0.85 mg mL^−1^) and stirred at room temperature for 12 h to form a PDA shell on the surface of A151/PEI complex (A151/PEI@PDA, AP). After the reaction, the AP solution was placed in a 30 kD ultrafiltration tube, and centrifuged at 12 000 rpm for 10 min. The precipitation was suspended in ultrapure water and washed three times. The N/P ratio was calculated according to the following formula: N/P = *m*_PEI_/*m*_DNA_ × 7.55.

### Preparation of APTS

NH_2_‐PEG_5000_‐TP was prepared as previously reported.^[^ ^14^ ^]^ Briefly, NH_2_‐PEG_5000_‐Mal (3 mg) and TP peptide (1 mg) were dissolved in 2 mL PBS (pH 7.2–7.4) and stirred overnight at room temperature under nitrogen protection. Then NH_2_‐PEG_5000_‐TP (10 mg) and NH_2_‐PEG_5000_‐SA (10 mg) were mixed with AP in Tris‐HCl buffer (pH 8.5, 10 mm) and stirred for 24 h at room temperature. Finally, APTS was obtained by centrifugation at 12 000 rpm for 10 min.

### Animal Care

Ethical statement: C57BL/6 mice (male, ≈20–25 g) were purchased from Si Pei Fu (Beijing) Biotechnology Co. Ltd. All procedures were approved by the Life Sciences Ethical Review Committee of Zhengzhou University. All mice were maintained under specific pathogen­free conditions with a 12 h light/12 h dark cycle. The accreditation number of the animal laboratory is SCXK (YU) 2018‐0004.

### Statistical Analysis

The experiment results were evaluated at least three times and presented as the mean ± standard deviation (SD). The statistical significance was performed by Student's *t*‐test, two‐tailed Student's *t*‐test, one‐way analysis of variance (ANOVA) with Tukey's post‐test, and two‐way ANOVA with Dunnett's post‐test. Differences were ranked significant when *p < 0.05, **p < 0.01, ***p < 0.001.

## Conflict of Interest

The authors declare no conflict of interest.

## Author Contributions

J.S. and N.Y. conceived the project. N.Y., W.W., and F.P. performed the experiments and analyzed the data. N.Y., Y.Z.Z., C.L., M.G., and Z.W. wrote the manuscript., K.Z., J.L., Z.Z., Y.Z., and J.S. supervised the project.

## Supporting information

Supporting Information

## Data Availability

The data that support the findings of this study are available in the supplementary material of this article.
