# Supplementary material for: A Neutrophil Hijacking Nanoplatform Reprograming NETosis for Targeted Microglia Polarizing Mediated Ischemic Stroke Treatment
Source: Adv Sci (Weinh). 2024 Mar 5;11(17):2305877. doi: 10.1002/advs.202305877 (PMC11077645; doi:10.1002/advs.202305877)
Supplement: Supplementary file 1 — Supporting Information [file ADVS-11-2305877-s001.pdf]

ADVANCED SCIENCE
Open Access

Supporting Information

for *Adv. Sci.*, DOI 10.1002/advs.202305877

A Neutrophil Hijacking Nanoplatform Reprograming NETosis for Targeted Microglia Polarizing Mediated Ischemic Stroke Treatment

Na Yin, Wenya Wang, Fei Pei, Yuzhen Zhao, Changhua Liu, Mingming Guo, Kaixiang Zhang, Zhenzhong Zhang, Jinjin Shi, Yun Zhang, Zhi-Hao Wang* and Junjie Liu**

Supplementary Materials for

A neutrophil hijacking nanoplatform reprograming NETosis for targeted microglia polarizing mediated ischemic stroke treatment

Na Yin, Wenya Wang *et al.*

*Corresponding author. Email: zhangyun@zzu.edu.cn (Y.Z.). wangzhihao@zzu.edu.cn (Z.W.). liujunjie@zzu.edu.cn (J.L.)

This PDF file includes:

Supplementary Text
Figs. S1 to S27

Supplementary Text

Materials and methods

A151 (5'-TTAGGGTTAGGGTTAGGGTTAGGG-3') was purchased from Tsingke Biotechnology Co., Ltd. (Beijing, China). TP peptide (CGQLKHLEQQEGC) synthesized by Peptide Industry Biotechnology Co., Ltd. (Nanjing, China). LPS, DAPI, DCFH-DA, Calcein/PI Live/Dead assay kit and DiO were purchased from Beyotime Biotechnology (Shanghai, China). Neutrophil isolation kit and BCA protein assay kit were purchased from Solarbio (Beijing, China). Live body skull transparency kit was purchased from Javis Biomedical Co., Ltd. (Wuhan, China). FITC-dextran was purchased from Merck. Staurosporine, PMA, and fMLP were purchased from MedChemExpress. All chemicals were purchased from Sigma-Aldrich unless otherwise specified and were used as received. Myeloperoxidase antibody, STING antibody, NF-κB p65 antibody, which were purchased from Cell Signaling Technology; iNOS antibody, and cGAS antibody, were purchased from Abcam; Caspase-1 antibody and GAPDH antibody were purchased from Proteintech. Alexa Fluor® 488 anti-mouse CD206 was purchased from BioLegend. Ly6G Antibody and Sytox were purchased from Thermo Fisher Scientific.

Stability experiment

The nanoparticles were incubated in PBS (pH 7.4) or 10% FBS solution at specific time intervals, then was detected the particle size and zeta potential by a Malvern Zetasizer Instrument. The accelerated stability of nanoparticles was detected at 40°C and 75% relative humidity for 4 weeks.

The adhesion of neutrophils to bEnd.3 cells

The adhesion of the activated neutrophils and unactivated neutrophils to bEnd.3 was evaluated by the flow chamber experiment. The bEnd.3 cells (2×10^5) were seeded in the microscope cover glass (Absin, 10 mm×10 mm, thickness: 0.17 mm). After the bEnd.3 cells were labeled with a green live cell tracer probe (MK, China), and the glass plates were fixed in the flow chamber. After 24 h, TNF-α (20 ng/mL) was added and incubated for 24 h to induce inflammation. Then, 1×10^6 neutrophils (labeled with red live cell tracker) were circulated in the flow chamber by a peristaltic pump (LONGER, BT100-2J), and the shear stress was selected at 1.25 dyn/cm^2. After 1 h, the cells were observed by CLSM (TCS SP8, Leica).

Evaluation of hitchhiking efficiency of neutrophils

Neutrophils were extracted from the blood of mice using a neutrophilic isolation kit. The neutrophils (1×10^6 cells/well) were seeded on 6-well plates. fMLP (1 μM) pretreatment was first

used to mediate neutrophil activation, then added RhB-labeled APS. They were incubated for 1 h under static and flow conditions. APS was mixed with neutrophils containing PBS (pH 7.4) solution, and then was injected to the flow chamber. After that, the flow chamber was connected to a peristaltic pump (LONGER, BT100-2J) using a silicone tube to provide the driving force for flow condition. The shear stress of peristaltic pump was 1.25 dyn/cm^2. After flowing for 1 h, the internalization of APS by neutrophils was analyzed by flow cytometry (FACSCanto II, BD).

Investigation of APTS hitchhiking efficiency

APTS deceleration enhanced neutrophil hitchhiking efficiency was evaluated by flow cytometry. fMLP (1 μM) pretreatment was first used to mediate neutrophil activation. Then, APS and neutrophils or APTS and neutrophils were circulated in a flow chamber inoculated with bEnd.3 for 1 h. Neutrophils were labeled with APC-conjugated CD11b antibody (1:100) and analyzed by flow cytometry.

Neutrophils migrating across BBB

5×10^5 cells/well neutrophils and APTS-neutrophils were inoculated into the upper chamber of the transwell insert, and fMLP was added to the lower chamber to induce neutrophil migration. After 4 h, the neutrophils retained in the upper chamber of the transwell membrane were removed using a swab. Cells migrating to the surface of the lower membrane or the lower chamber were immobilized with paraformaldehyde and stained with 0.1% crystal violet. The number of migrating neutrophils was calculated.

BBB penetration *in vitro*

bEnd.3 cells (1×10^4 cells/well) were plated on the upper side of 0.4 μm pore size 24-well transwell membrane (Corning, USA) to construct an *in vitro* BBB model. When the cell resistance was greater than 200 Ω, APTS and APTS- neutrophils were added to the upper chamber. The fluorescence intensity of the lower chamber was measured by the fluorescence spectrophotometer.

NETs inhibition and death of neutrophils

The neutrophils (1×10^6 cells/well) were seeded on 6-well plates. After 30 min, PMA was induced (100 nM) for 1 h, and the cells were washed with PBS three times (pH 7.2-7.4). Then PBS, AP, and APTS in fresh culture media were added, respectively. After incubation for 4 h, the cells were fixed with 4% paraformaldehyde for 15 min. Then the cells were treated with immunostaining blocking solution containing Triton X-100 for 10 min. Next, primary antibodies including anti-MPO (1:200), anti-NE (1:200), and anti-H3Cit (1:200) antibodies were incubated with cells at 4°C

overnight and incubated with Alexa Fluor 488 or 594-labeled secondary antibodies at room temperature for 1 h. Finally, the expression of MPO, NE, and H3Cit in neutrophils of different treatment groups was observed by CLSM. In addition, sytox was used to detect extracellular DNA release, and apoptosis was detected by living cell Caspase-3 and Annexin V apoptosis detection kits.

Extraction of ABs

ABs were collected by differential centrifugation[1]. Briefly, the apoptotic neutrophil culture medium of different treatment groups was collected and centrifuged at 200 g for 10 min to remove cells and debris. The supernatant was further centrifuged at 2000 g for 20 min to concentrate the ABs. Then, ABs were washed with PBS (pH 7.4) three times and stored at -80°C for subsequent experiments.

Specific uptake of ABs by microglia

To verify the specific uptake of ABs by microglia in vitro, DiO-labeled ABs were added to bEnd.3, PC12, CTX, and BV2 cells incubated for 4 h. After incubation, the cytoskeleton was stained with phalloidin, the nucleus was labeled with DAPI, and the uptake of ABs by different cells was observed by CLSM.

Microglia polarization

BV2 cells were seeded into a confocal culture dish at a density of 1×10^4 cells/well, and induced with LPS (1 μg/mL) for 12 h. After that, 2 μg/mL poly (dA:dT), PBS, ABs, and ABs-APTS were added to the media for 4 h. BV2 cells (1×10^4 cells/well) were seeded in a confocal culture dish. After refreshing the medium, the cells were incubated for another 36 h. Then the cells were fixed with 4 % formaldehyde at room temperature for 15 min. Furthermore, the cells were treated with immunostaining blocking solution containing Triton X-100 for 10 min. Next, the cells were stained iNOS and CD206 overnight at 4°C, followed by counterstaining with DAPI for 10 min. Fluorescence imaging was performed using CLSM. The levels of TNF-α, IL-6, Arg-1, and IL-10 in cell culture supernatant were measured using ELISA kits.

The cell supernatants of different treatment groups were collected, and the changes of pro-inflammatory cytokines IL-6, TNF-α, and anti-inflammatory cytokines IL-10 and Arg-1 were detected by ELISA detection kit.

Investigation of neuroprotection *in vitro*

100 nM PMA-induced neutrophils (PMA-neutrophile) and APTS-treated PMA-neutrophils (5×10^5 cells/well) were inoculated into the upper chamber of the transwell plate. PC12 cells (3×10^5/well) were seeded in the lower chambers. An oxygen-glucose deprivation (OGD) model was established by placing 12 well transwell plates in an AnaeroPack (MGC, Japan). The OGD model was treated for 24 h followed by reoxygenation for 12 h. Cell viability was assessed by Live & Dead assay kit.

MCAO model

Ischemic stroke was induced by the transient middle cerebral artery occlusion (MCAO) model in mice [2]. Mice were first anesthetized with 3% isoflurane. The anesthetized mice were then transferred to a thermostatic operating table and the isoflurane maintenance concentration was adjusted to 1.5%. Cut the median skin of the neck, the common carotid artery (CCA), internal carotid artery (ICA), and external carotid artery (ECA) were carefully separated under the stereomicroscope. Then a silicon suture with a diameter of 0.22 mm (Beijing Cinontech Co. Ltd.) blocked the blood supply of the middle cerebral artery (MCA) by inserting the ECA. After 90 minutes of occlusion, the silicon suture was removed to restore MCA blood flow. Mice in the sham operation group received the same anesthesia and arterial exposure procedure, but no suture insertion.

***In vivo* multiphoton microscopy**

The mice were anesthetized with 1.5% isoflurane. After fixation in a custom-made head holder, the skull was made transparent using a live skull Transparency kit. Mice were intravenously injected with PE-Ly6G antibody (3 μg). Blood vessels were visualized by intravenous injection of FITC-dextran (2,000,000 Da, 0.1 mL of 10 mg/mL). After 30 min, the adhesion and extravasation of neutrophils on blood vessels in the brains of ischemic mice were further observed using in vivo multiphoton microscopy.

Light-sheet microscopy: After injection of RhB-APS or APTS for 40 min, mice were intravenously injected with FITC-dextran (2,000,000 Da, Sigma-Aldrich, 0.1 mL of 10 mg/mL) to label blood vessels. After 30 min, the brain tissue was taken out and treated according to the transparent brain kit (PEGASOS) and observed under a light-sheet microscope.

Biodistribution of APTS

AP, APS, and APTS were labeled with RhB. MCAO model mice were randomly divided into 3 groups, 3 mice in each group. A total of 200 μL of free RhB and RhB-labeled different preparations were injected into MCAO mice through the tail vein. After 12 h, the brain, heart, liver, spleen,

lung, and kidney were collected and observed under the IVIS system. The brain tissue was further cut into 5 uniform slices with a thickness of 2 mm and placed in a small animal living imager for observation and photo recording.

Treatment

MCAO mice were randomly divided into 7 groups: PBS, Lip/A151, AP, PDATS, AP, APT, APS, and APTS groups. Different formulations were injected into the mice through the tail vein at 0, 2, 4, and 6 days after MCAO surgery. The dosage of PDA was calculated as 10 mg/kg.

Neurological deficit score

After the model mice were treated with different formulations, the well-established five-point scale methodology was performed for the neurological deficit score (Rating scale, 4 = spontaneous circling, 3 = circling to left by pulling the tail, 2 = decreased grip strength of left forepaw, 1 = failure to extent left forepaw, and 0 = no deficit).

Behavior tests

For the adhesive test, the mice were trained 3 days before MCAO. Specifically, a sticker of 3×3 mm^2 was placed on the paralyzed forepaw of mice. Then record the time when the mouse perceived the presence and successful removal of the stick. After the MCAO model, the mice were tested on days 3, 5, and 7. If the mouse failed to remove the stick within 120 s, the time was recorded as 120 s and the experiment was terminated. Morris water maze was used to record the swimming swim path trajectories of mice on day 7.

TTC staining

After treatment, the mice were sacrificed and the brain tissue was removed. The brain tissue was immediately frozen at -80°C for 5 min, cut into 2 mm wide coronal sections, and stained with 2% 2,3,5-triphenyltetrazolium chloride (TTC) phosphate buffer at 37ºC for 20 min.

Hematoxylin-eosin (H&E) staining

Mice brain tissues were fixed with 4% paraformaldehyde for 24 h, dehydrated and embedded, and tissue wax blocks were made. The wax blocks were processed by hematoxylin-eosin (H&E) staining. The pathological changes and neuronal damage were analyzed by observing the cell morphology of each group.

BBB protection *in vivo*

2% Evans Blue (EB) saline solution was injected into mice via the tail vein at a dose of 4 mg/kg. After 2 h, the mice were perfused with PBS to collect brain tissue. The injured brain tissue was

homogenized and centrifuged at 12000 rpm for 20 min to collect the supernatant. The absorbance of the supernatant at 620 nm was measured by a microplate reader to detect the concentration of EB in the brain.

Immunofluorescent staining

The brain sections were processed for immunofluorescent staining using primary antibodies: anti-Ly6G antibody for neutrophils, anti-H3Cit antibody for NETs, anti-iNOS antibody, and anti-CD206 antibody. ImageJ software was used to quantify the fluorescence intensity of all images for further analysis.

Measurement of brain cytokines

The harvested brain tissue was homogenized with saline and centrifuged at 12000 rpm for 5 min. The TNF-α, IL-10, Arg-1, and IL-6 levels in each sample were analyzed by TNF-α ELISA kits (Proteintech, Wuhan, China), IL-10 ELISA kits (Proteintech, Wuhan, China), Arg-1 ELISA kits (Cloud-Clone, Wuhan, China), and IL-6 ELISA kits (Boster, USA) according to the manufacturer's instructions.

References

[1] G. Dou, R. Tian, X. Liu, P. Yuan, Q. Ye, J. Liu, S. Liu, J. Zhou, Z. Deng, X. Chen, S. Liu, Y. Jin, Sci Adv 2020, 6, eaba2987.

[2] J. Yuan, L. Li, Q. Yang, H. Ran, J. Wang, K. Hu, W. Pu, J. Huang, L. Wen, L. Zhou, Y. Jiang, X. Xiong, J. Zhang, Z. Zhou, ACS Nano 2021, 15, 16076.

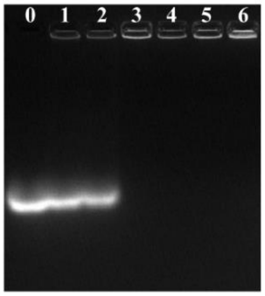

Fig. S1. A151/PEI gel retardation assay with different nitrogen to phosphate (N/P) ratio. (0): A151; (1) A151:PEI=1:1; (2) A151:PEI=1:2; (3) A151:PEI=1:3; (4) A151:PEI=1:4; (5) A151:PEI=1:5; (6) A151:PEI=1:6.

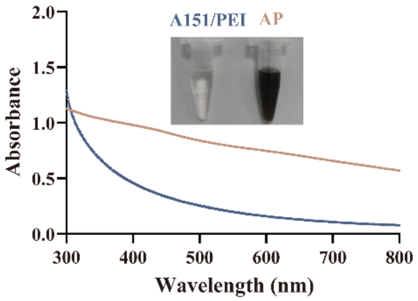

Fig. S2. UV-vis absorption spectra and images of A151/PEI and AP.

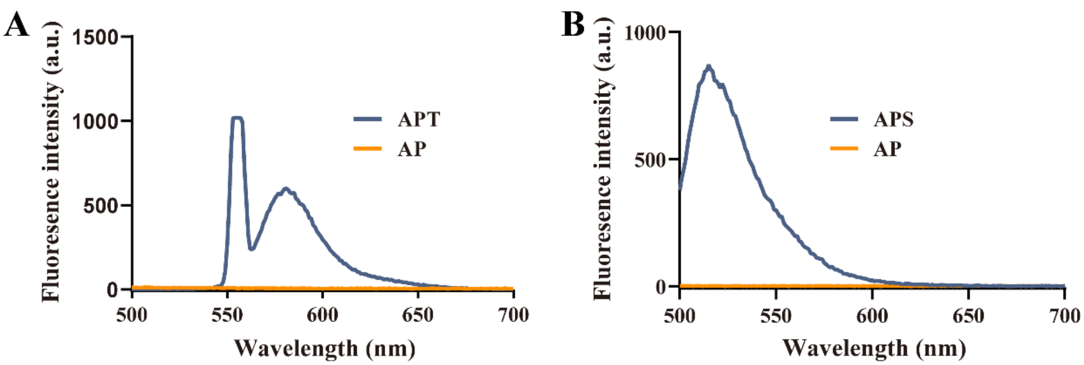

Fig. S3. The modification of TP peptide and SA on the surface of PDA. (A) Fluorescence intensity of AP and AP modified by TP peptide (Rhodamine B-labeled TP peptide). (B) Fluorescence intensity of AP and AP modified by SA (FITC-labeled SA).

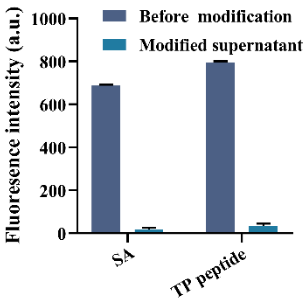

Fig. S4. Changes of fluorescence intensity of FITC labeled TP and SA before and after modification. Data are presented as mean ± SD (n = 3).

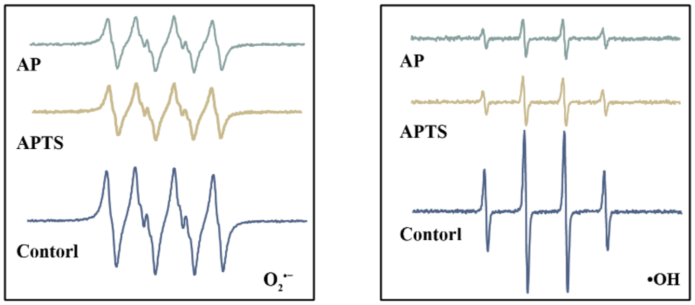

Fig. S5. Electron spin resonance spectrum of AP and APTS.

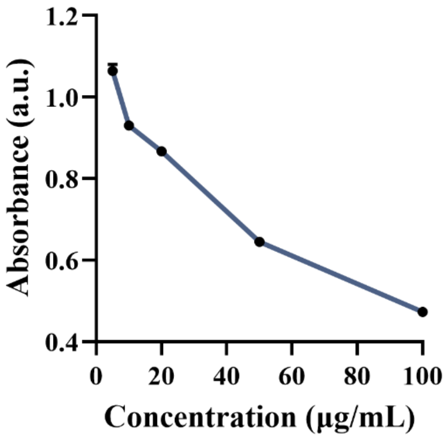

Fig. S6. The absorbance of H_2O_2 after treatment with different concentrations of APTS. Data are presented as mean ± SD (n = 3).

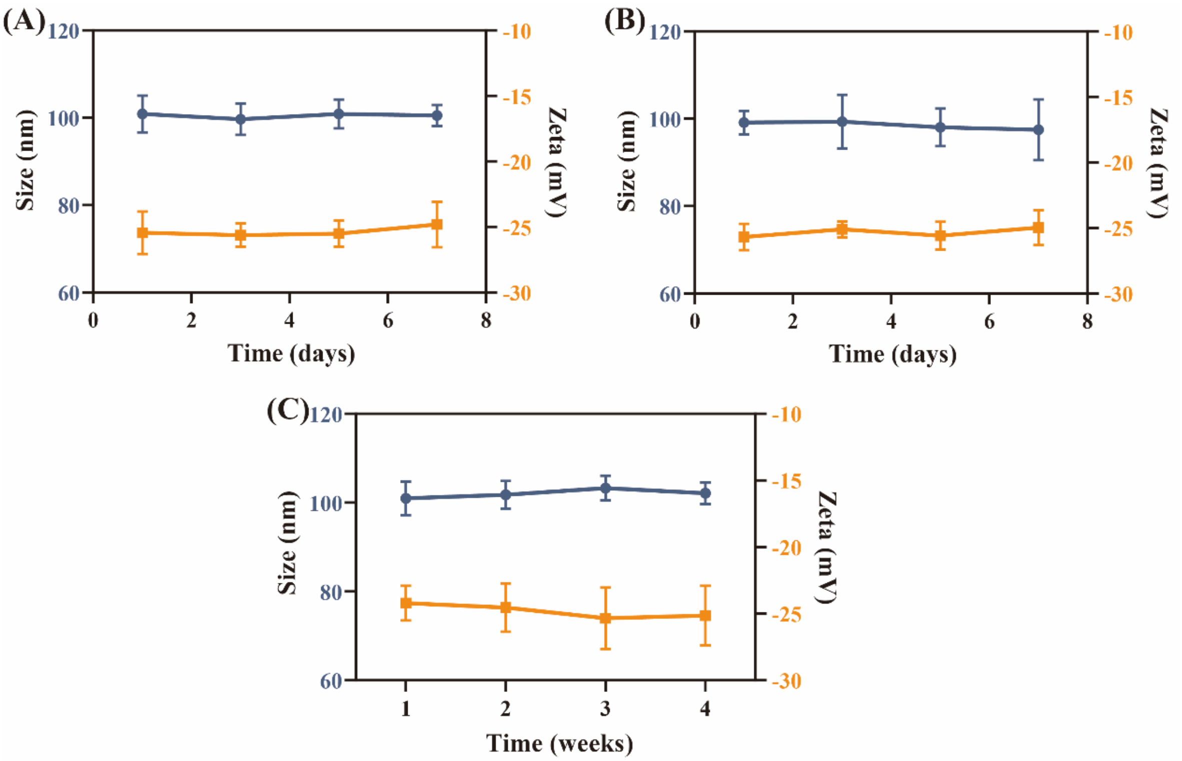

Fig. S7. Particle size and zeta potential of APTS in PBS (A) and 10% FBS (B) solution. (C) Particle size and zeta potential of APTS during accelerated stability experiment. Data are presented as mean ± SD (n = 3).

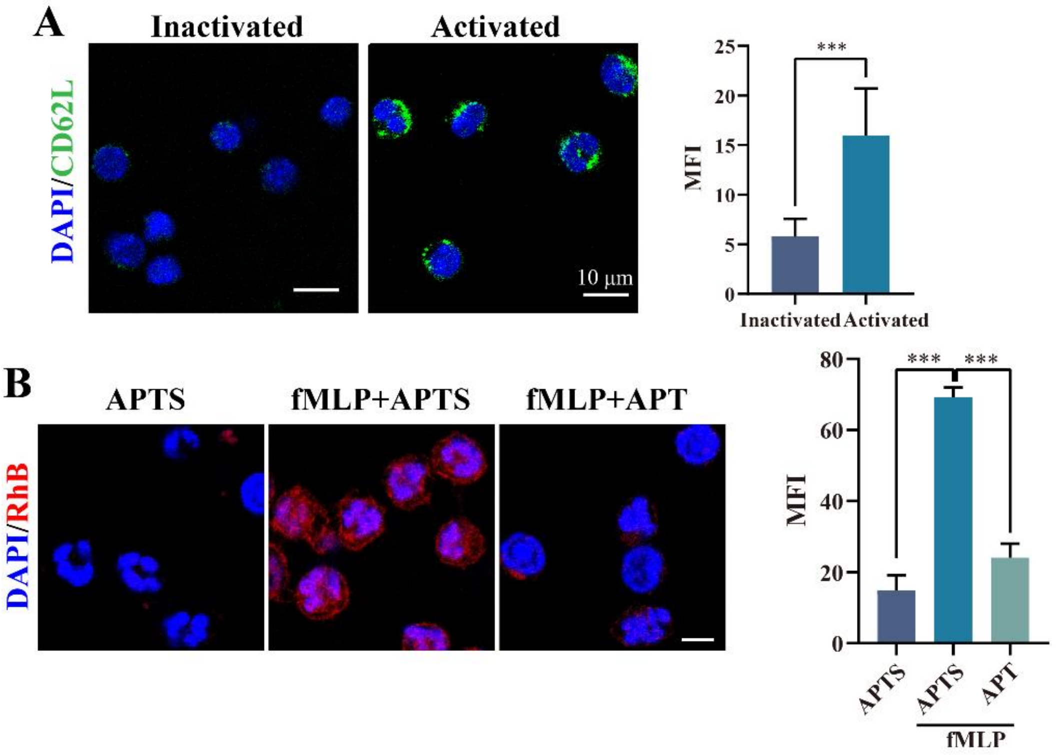

Fig. S8. L-selectin expression on activated or inactivated in peripheral blood neutrophil membranes. (A) Confocal laser scanning microscopy (CLSM) images of cells stained with PE anti-mouse CD62L antibody for L-selectin (green) and DAPI for nuclei (blue). (B) Cellular uptake of APTS and APT at 2 h were characterized in neutrophils and activated neutrophils (fMLP-treated). APTS and APT were labeled with RhB (red). Scale bar: 5 μm. Quantitative analysis of APTS and APT uptake in different treatments. Data are presented as mean ± SD (n = 6). Statistical significance was calculated by one-way ANOVA. ***$P < 0.001$.

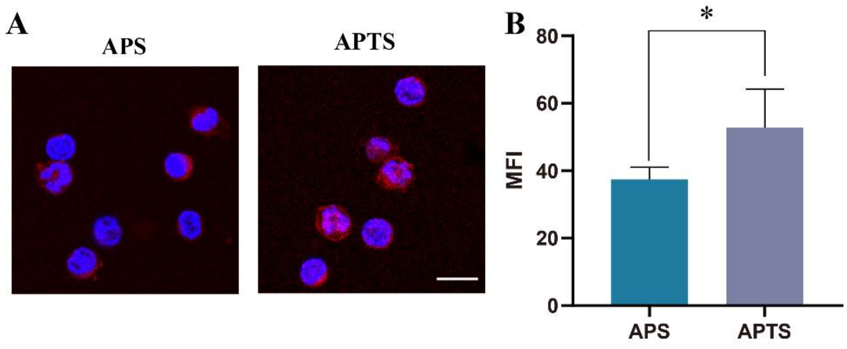

Fig. S9. The confocal laser scanning microscopy (CLSM) images of neutrophils incubated with different nanoparticles under flow condition. Data are presented as mean ± SD (n = 5). Statistical significance was calculated by one-way ANOVA. *$P < 0.05$.

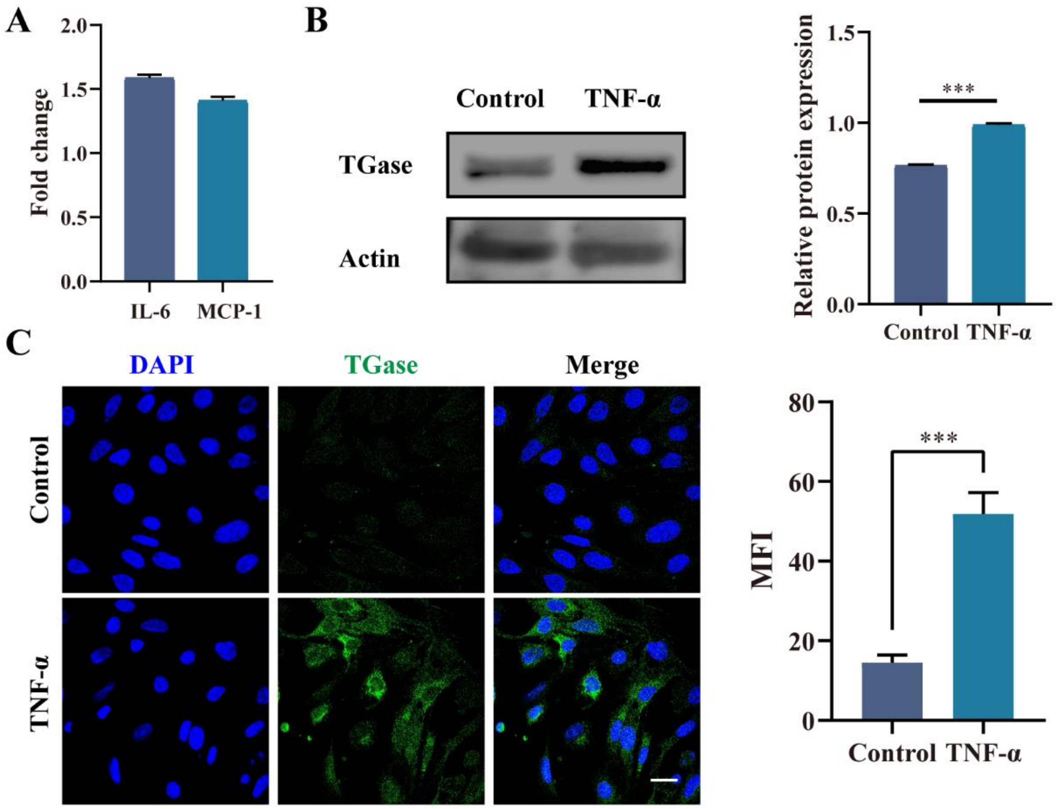

Fig. S10. Expression of TGase in inflammatory endothelial cells. (A) Relative expression of IL-6 and MCP-1 of bEnd.3 cells (data presented as mean ± SD, n = 3). (B) Western blot and quantification of TGase in control and TNF-α groups. Results are expressed as the mean ± SD (n = 3). (C) Represented immunostaining images of TGase in bEnd.3 cells after different treatments (bEnd.3 cells were induced by 20 ng mL^{-1} TNF-α for 24 h). Scale bar: 25 μm. Mean fluorescence intensity (MFI) statistics of immunofluorescent staining images (n = 6). Data are presented as mean ± SD. Statistical significance was calculated by student's t-test. ***$P < 0.001$.

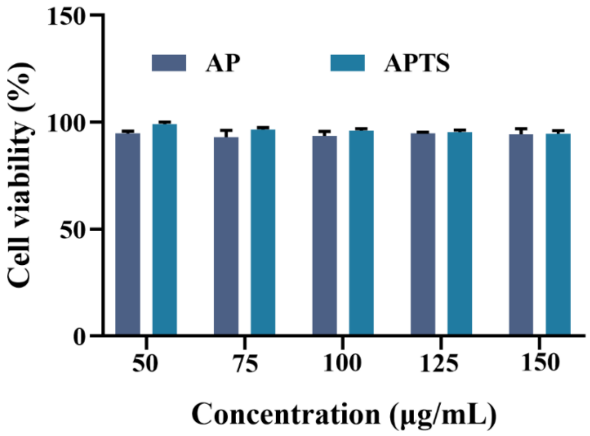

Fig. S11. Neutrophil viability treated with different concentrations of AP and APTS. Data are presented as mean ± SD (n = 4).

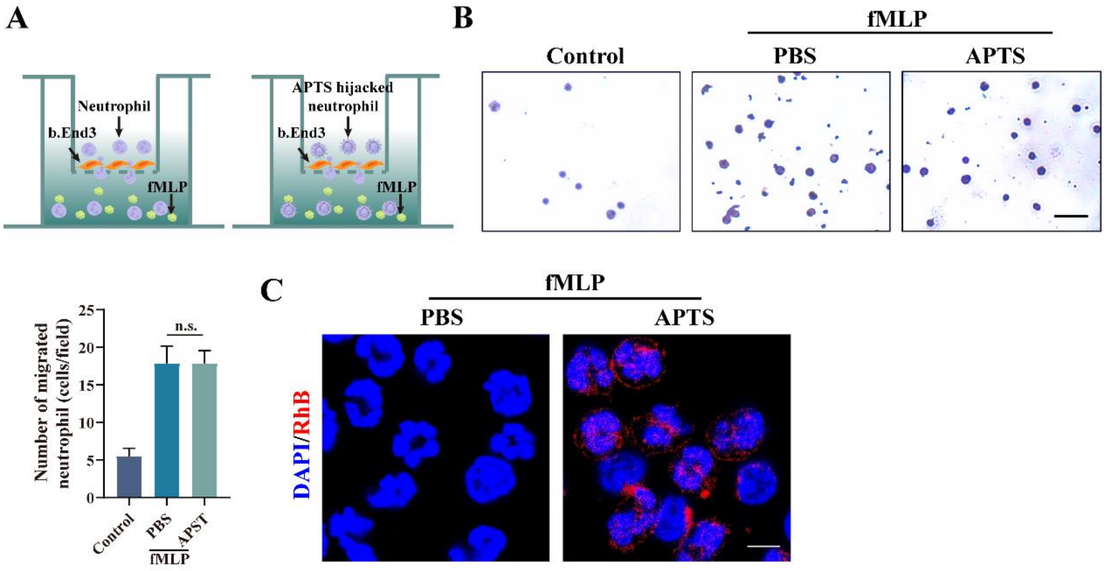

Fig. S12. Migration ability of APTS-hijacked neutrophils. (A) Diagram of the in vitro model to evaluate neutrophils migration capability across BBB using a transwell assay. (B) Representative images of the migrated (in purple) through the BBB; Scale bar: 200 µm. Quantitative analysis of the in vitro migration as illustrated in (B). Data are presented as mean ± SD (n = 6). (C) CLSM images of neutrophils (Nuclei, DAPI, blue; APTS, RhB, red). Scale bar: 5 µm. Statistical significance was calculated by one-way ANOVA. ***P < 0.001.

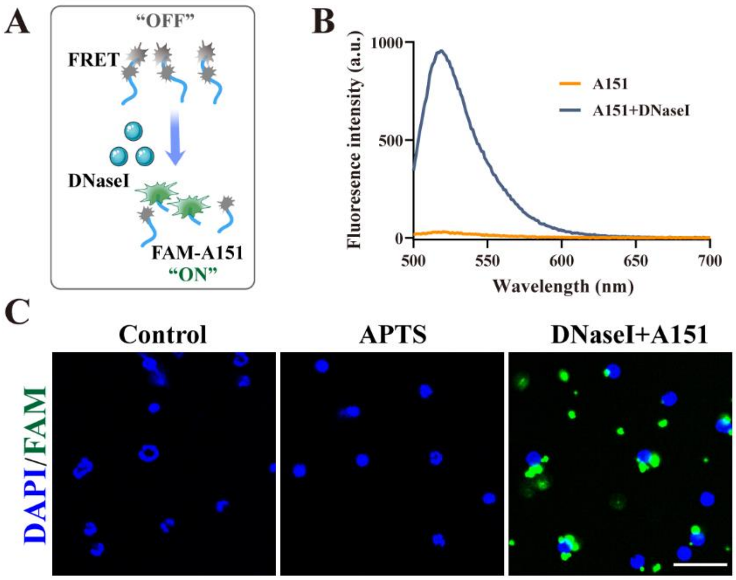

Fig. S13. A151 stability. (A) Illustration of FRET effect under DNaseI treatment. (B) Fluorescence intensity of A151 before and after DNaseI addition. (C) Confocal fluorescence images of APTS for detection of A151 stability in neutrophils. DNaseI + A151 as a positive control. Scale bar: 25 μm.

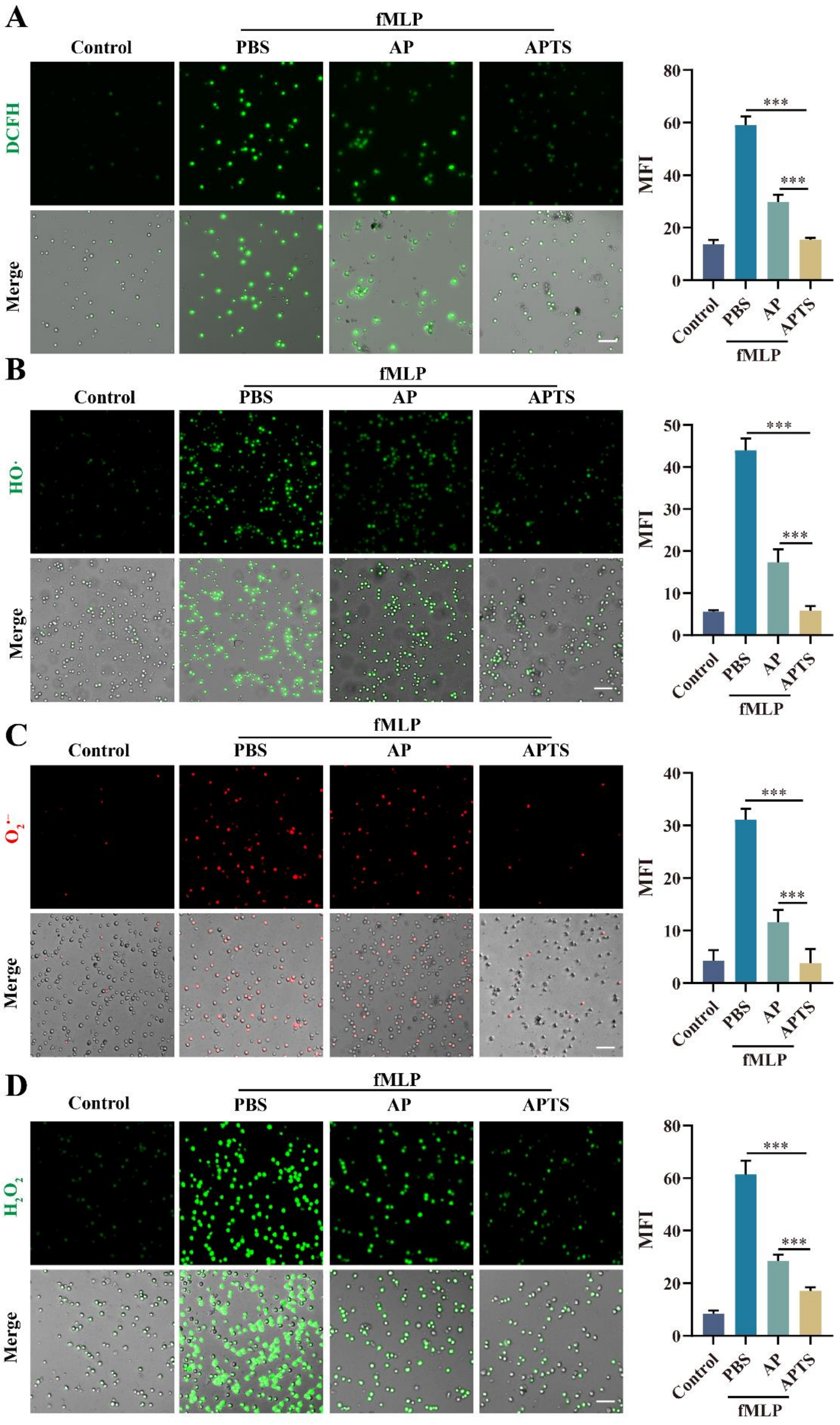
A
B
C
D
fMLP
Control
PBS
AP
APTS
DCFH
HO·
O2·−
H2O2
Merge
MFI
***

Fig. S14. ROS scavenging in neutrophils. (A to D) Fluorescence imaging of total ROS, $\cdot$OH, $O_2^{\bullet-}$, and H_2O_2 in neutrophils with different treatments (Scale bar: 25 μm.) as well as MFI of A to D in neutrophils with different treatments. Data are presented as the mean ± SD (n = 6). Statistical significance was calculated by one-way ANOVA. ***P < 0.001.

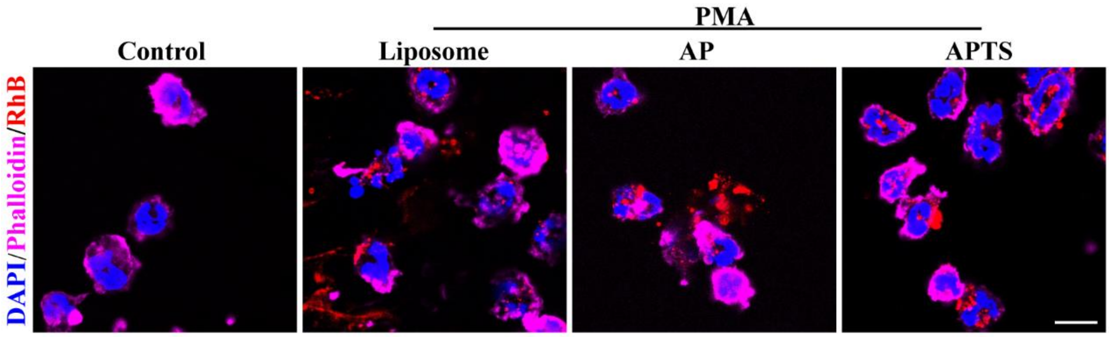

Fig. S15. Distribution of nanoparticles in neutrophils. Neutrophils were induced by 100 nM PMA for 4 h, and then treated with PBS, AP, and APTS. Scale bar: 10 μm.

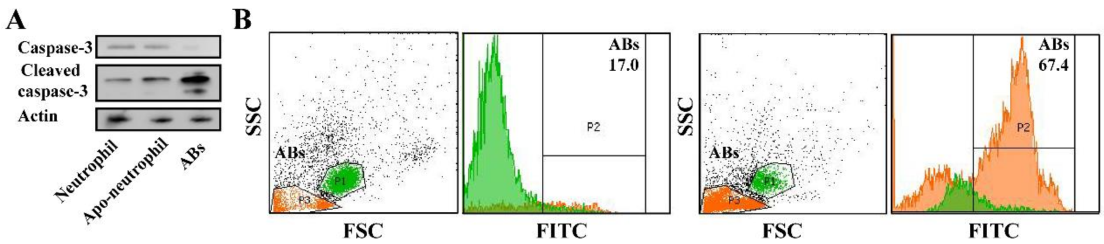

Fig. S16. (A) Western blot analysis of caspase-3, and cleaved caspase-3 protein expression in neutrophil, Apo-neutrophil (apoptotic neutrophil), and ABs. (B) Flow cytometric analysis of ABs (yellow) and neutrophils (green) stained with Annexin V- FITC.

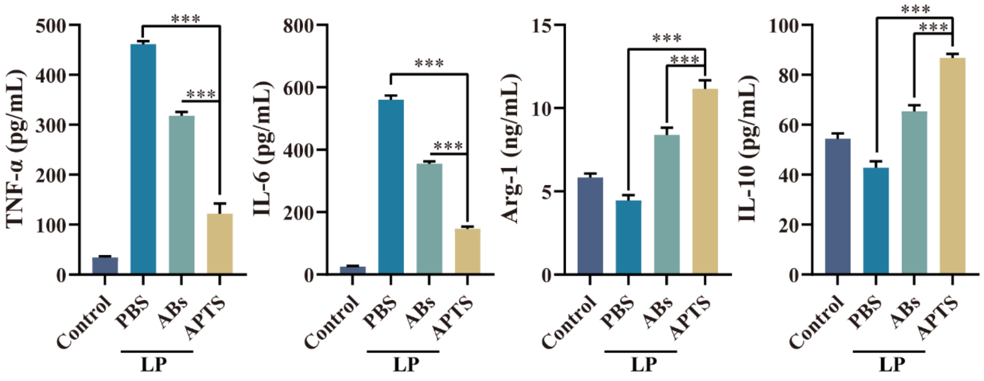

Fig. S17. Detection of the levels of cytokines in the supernatant (n = 3). Data are presented as mean ± SD. Statistical significance was calculated by one-way ANOVA. ***$P < 0.001$.

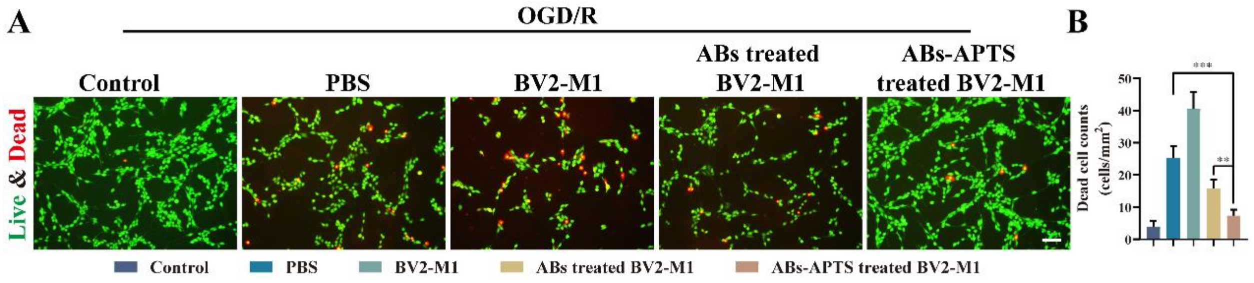

Fig. S18. Live & Dead staining for PC12 cells receiving different treatments. Scale bar: 200 μm; Quantification of dead cell numbers in different treatments (n = 6). Data are presented as mean ± SD. Statistical significance was calculated by one-way ANOVA. $^{**}P < 0.01$, $^{***}P < 0.001$.

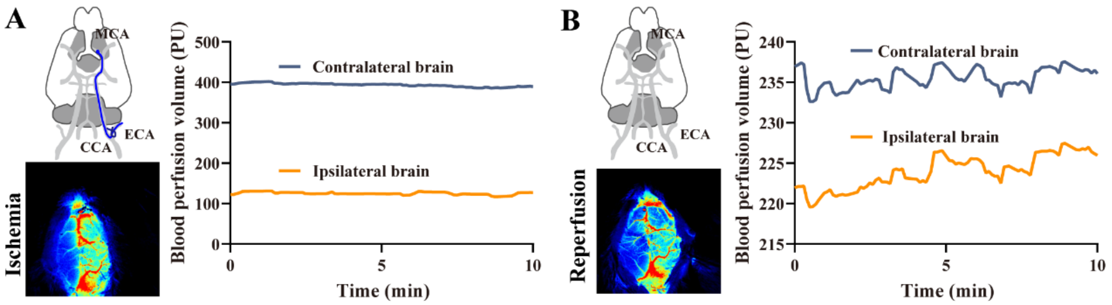

Fig. S19. Laser speckle analysis after middle cerebral artery occlusion and reperfusion.

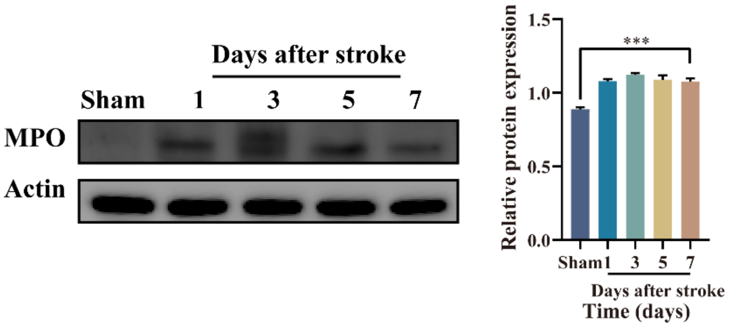

Fig. S20. Western blot analysis of MPO (left). Data are presented as mean ± SD (n = 3). Statistical significance was calculated by one-way ANOVA. ***P < 0.001.

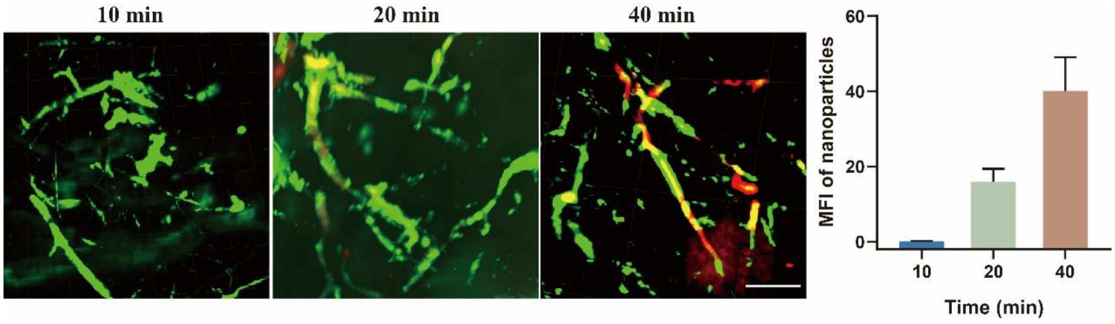

Fig. S21. Representative light-sheet microscope images of the distribution of nanoparticles at the vascular site over time and corresponding quantitative fluorescence analysis (n = 6). Green: FITC dextran-labeled blood vessels; Red: RhB-labeled APS or APTS. Scale bar: 200 μm. Data are presented as mean ± SD.

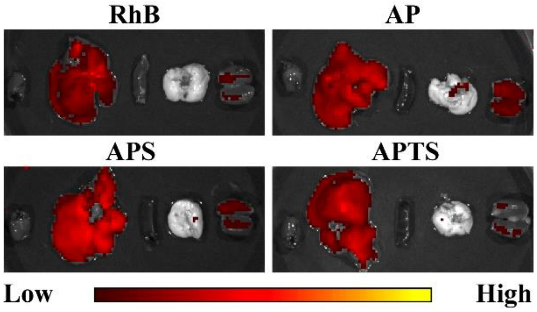

Fig. S22. Representative *ex vivo* fluorescence images of major organs dissected from MCAO mice at 12 h after intravenous injection (*i.v.*) with AP, APS, or APTS.

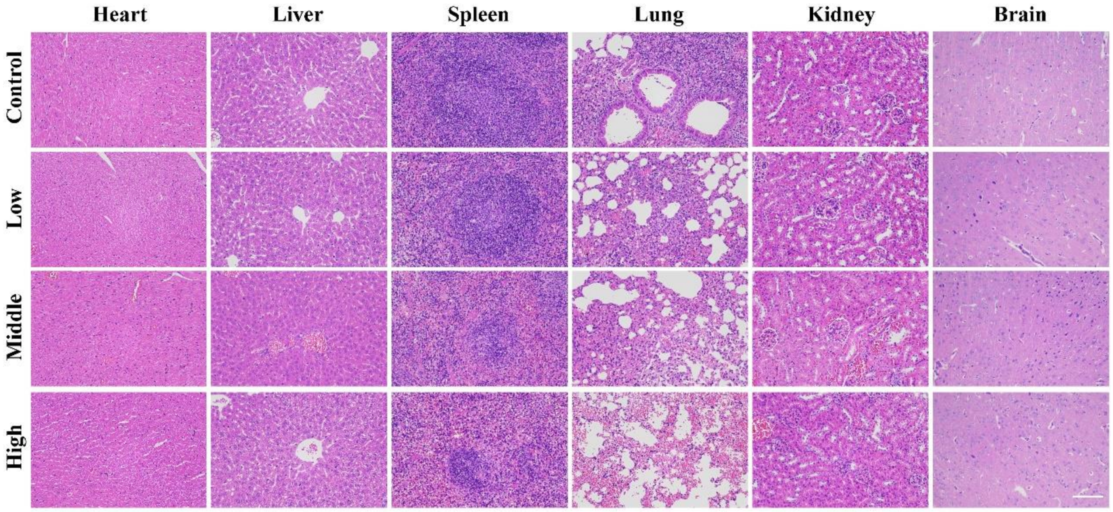

Fig. S23. The heart, liver, spleen, lung, kidney, and brain of mice were stained with H&E after 14 days of intravenous administration of 5, 10, and 15 mg/kg APTS. PBS intravenous injection as a control group. Scale bar: 100 μm.

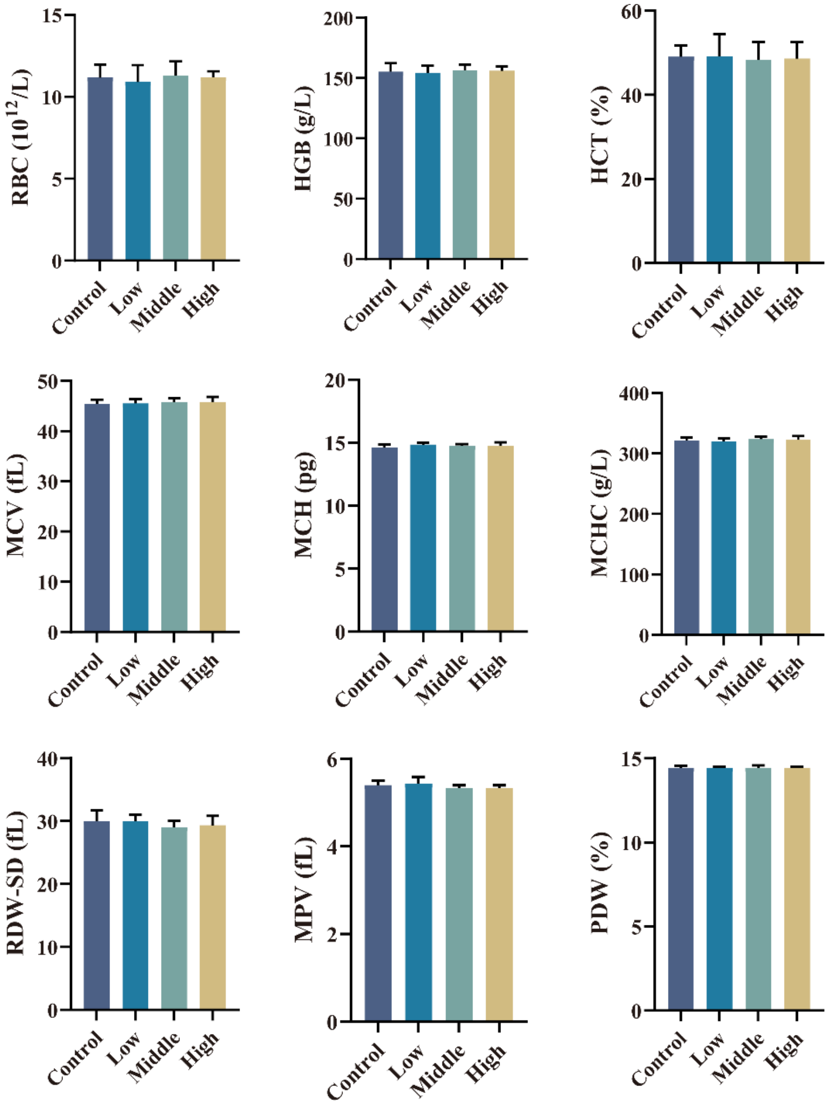

Fig. S24. The changes of routine blood parameters in mice after intravenous injection of 5, 10, and 15 mg/kg APTS for 14 days. PBS intravenous injection as a control group. Red blood cells (RBC), Hemoglobin (HGB), Hematocrit (HCT), Mean corpuscular volume (MCV), Mean corpuscular hemoglobin (MCH), Mean corpuscular hemoglobin concentration (MCHC), Red blood cell distribution width-standard deviation (RDW-SD), Mean platelet volume (MPV) and Platelet distribution width (PDW). Data are presented as mean ± SD (n = 3).

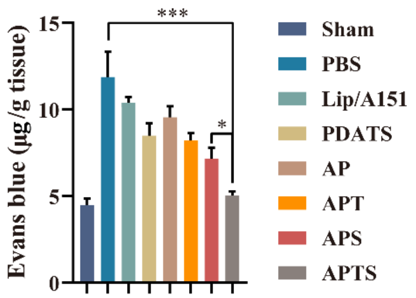

Fig. S25. Evans Blue (EB) (2%, 4 mg/kg) was administered intravenously to mice. After 2 h, the brain tissue of sacrificed mice was homogenized and EB concentration was measured at 620 nm using a microplate reader. Data are presented as mean ± SD (n = 3). Statistical significance was calculated by one-way ANOVA. *P < 0.05, ***P < 0.001.

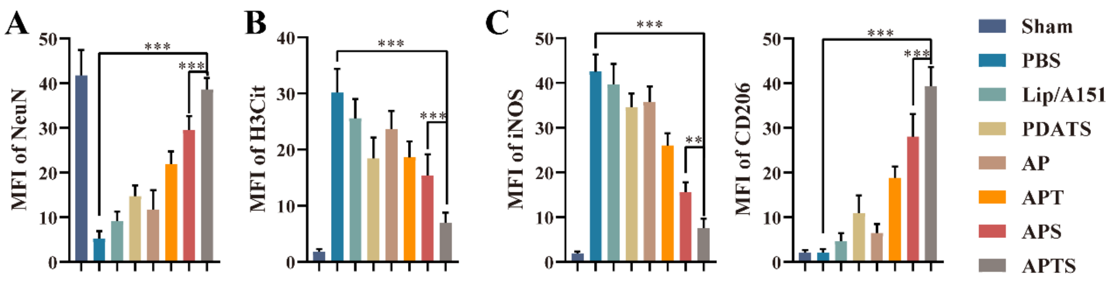

Fig. S26. (A-C) Quantification of the NeuN, H3Cit, CD206, and iNOS. Data are presented as mean ± SD (n = 3). Statistical significance was calculated by one-way ANOVA. $**P < 0.01$, $***P < 0.001$.

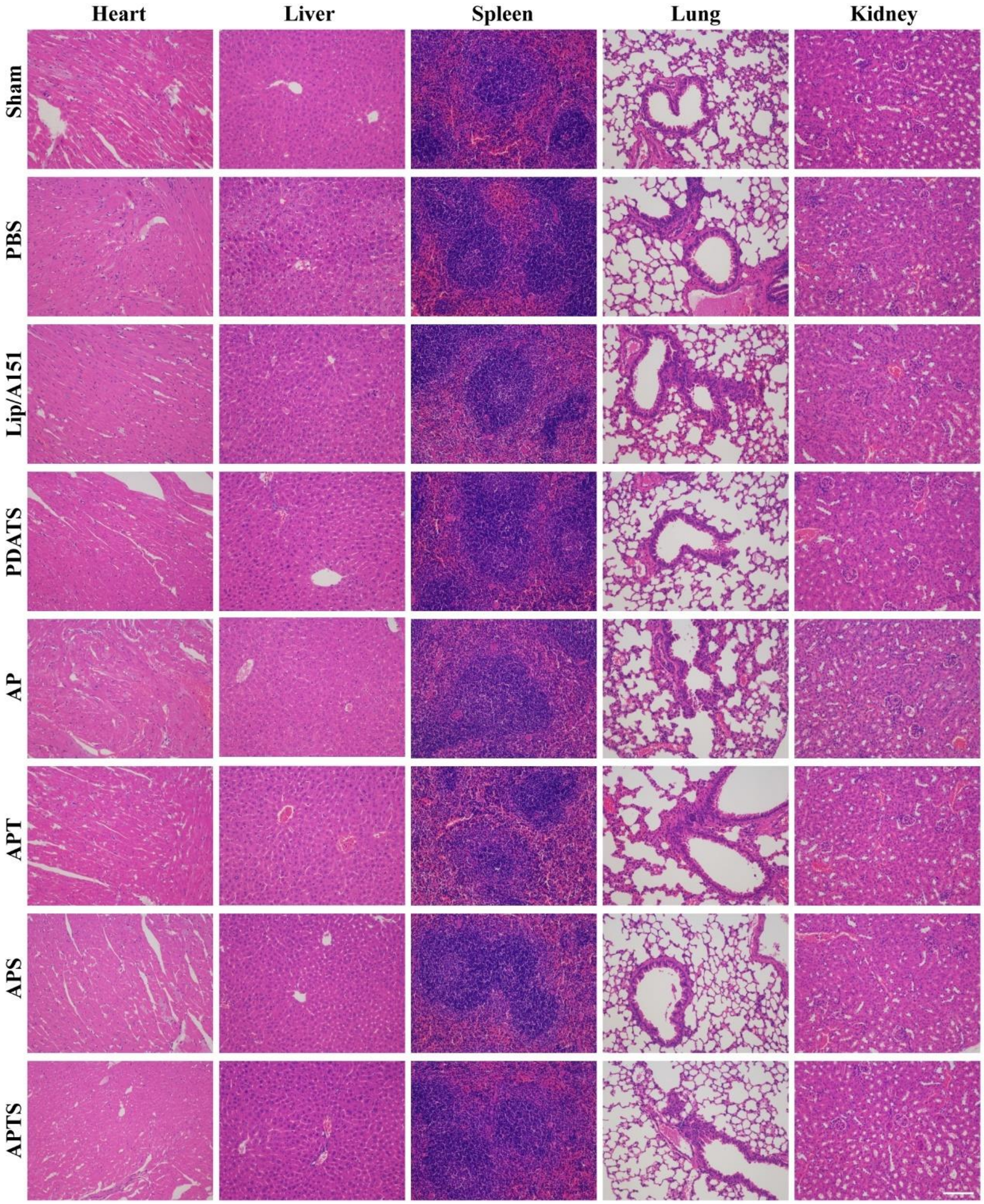

Fig. S27. H&E staining of different groups from heart, liver, spleen, lung, and kidney. Scale bar: 100 μm.
